# Molecular Characterization of the α-Subunit of Na^+^/K^+^ ATPase from the Euryhaline Barnacle *Balanus improvisus* Reveals Multiple Genes and Differential Expression of Alternative Splice Variants 

**DOI:** 10.1371/journal.pone.0077069

**Published:** 2013-10-09

**Authors:** Ulrika Lind, Magnus Alm Rosenblad, Anna-Lisa Wrange, Kristina S. Sundell, Per R. Jonsson, Carl André, Jonathan Havenhand, Anders Blomberg

**Affiliations:** 1 Department of Chemistry and Molecular Biology, University of Gothenburg, Gothenburg, Sweden; 2 Department of Biological and Environmental Sciences, University of Gothenburg, Gothenburg, Sweden; St. Georges University of London, United Kingdom

## Abstract

The euryhaline bay barnacle *Balanus improvisus* has one of the broadest salinity tolerances of any barnacle species. It is able to complete its life cycle in salinities close to freshwater (3 PSU) up to fully marine conditions (35 PSU) and is regarded as one of few truly brackish-water species. Na^+^/K^+^ ATPase (NAK) has been shown to be important for osmoregulation when marine organisms are challenged by changing salinities, and we therefore cloned and examined the expression of different NAKs from *B. improvisus*. We found two main gene variants, *NAK1* and *NAK2*, which were approximately 70% identical at the protein level. The NAK1 mRNA existed in a long and short variant with the encoded proteins differing only by 27 N-terminal amino acids. This N-terminal stretch was coded for by a separate exon, and the two variants of *NAK1* mRNAs appeared to be created by alternative splicing. We furthermore showed that the two *NAK1* isoforms were differentially expressed in different life stages and in various tissues of adult barnacle, i.e the long isoform was predominant in cyprids and in adult cirri. In barnacle cyprid larvae that were exposed to a combination of different salinities and pCO_2_ levels, the expression of the long *NAK1* mRNA increased relative to the short in low salinities. We suggest that the alternatively spliced long variant of the Nak1 protein might be of importance for osmoregulation in *B. improvisus* in low salinity conditions*.*

## Introduction

Organisms living in fluctuating marine environments, such as estuaries and coastal waters, require special physiological adaptations. The capacity to not only withstand, but also to grow and reproduce in such fluctuating environments, may prove advantageous in the face of increased stress from climate-related changes projected for the coming century [1]. Barnacles (subphylum Crustacea; infraclass Cirripedia) are among the most obvious examples of tolerant species in such environments: they are sessile macro-invertebrates, commonly found along rocky shores in coastal areas worldwide, where they are exposed to a broad range of environmental conditions including substantial changes in salinity, temperature, desiccation and wave action. The euryhaline bay barnacle *Balanus improvisus* Darwin, 1854 (=*Amphibalanus improvisus*) is an extreme performer with regard to salinity-tolerance: both adults and larvae can thrive in close to freshwater (3 PSU) up to fully marine conditions (35 PSU) [2-4] and can survive fluctuating salinities [5]. *B. improvisus* is a hermaphrodite that produces free-swimming larvae, which go through several moults (six feeding nauplius stages followed by a non-feeding cyprid stage) before attaching to hard substrates and developing into adults. Although much is known about the ecology of *B. improvisus*, the physiological/molecular mechanisms responsible for its exceptional tolerance to salinity changes throughout the life cycle are not well understood. 

When exposed to different salinities, marine organisms either osmoconform, i.e. allow the osmolality of the extracellular fluids to equal that of the surrounding medium, or osmoregulate and maintain the extracellular fluids at a constant osmolality compared to the external environment. *B. improvisus* has been suggested to osmoconform at high salinities, but to osmoregulate at lower salinities to keep the hemolymph hyperosmotic to the surrounding water [4]. Early work hypothesized that active uptake of ions is the primary mechanism for osmoregulation in adult barnacles, particularly at low salinities [4]. It is well established that the maintenance of a hyperosmotic extracellular fluid mainly is achieved by active ion-transport. For some crustaceans and most fish, Na^+^/K^+^-ATPases (NAKs) act as the main driving force for the active uptake of ions [6,7], while in barnacles the importance of this class of transporters has not been investigated. In addition, there are still no experimental data that firmly establish a primary tissue or site for osmoregulation in barnacles. In some other crustacean groups the thoracic appendages show enhanced ion transport including osmoregulatory activity [8]. It was recently reported that in adult *Balanus amphitrite* (=*Amphibalanus amphitrite*), silver-stained epithelia (suggesting high ion transport activity) were detected in the thoracic appendages (cirri and penis), and on the mantle epithelia [9]. Thus, these tissues could be of importance for osmoregulation in barnacles. It has further been suggested that during osmoconformation, *B. improvisus* regulates intracellular osmolality and volume by changing intracellular amino acid concentrations, especially proline [4]. 

The NAK pump consists of two essential subunits, α and β. In vertebrates, the Na^+^/K^+^ ATPase subunits can also form functional complexes with additional regulatory proteins [10]. The α-subunit is the catalytic subunit and contains binding sites for Na^+^, K^+^ and ATP. It comprises around 1,000 amino acids and is well conserved among species, in particular in the 10 membrane-spanning helices. The Na^+^/K^+^ ATPase is located at the basolateral membrane of epithelial cells in osmoregulating organs and extrudes three Na^+^ ions into the hemolymph/blood and imports two K^+^ ions at the expense of one molecule of ATP [6,11]. During salinity acclimatization, regulation of Na^+^/K^+^ ATPase expression has been shown to occur [6,7,12,13]. Increased expression of Na^+^/K^+^ ATPase in low salinity leads to increased absorption of sodium from the surrounding medium as well as absorption of chloride through other transporters [6]. However, Na^+^/K^+^ ATPase expression has also been shown to increase when freshwater-acclimated organisms are transferred to higher salinities [14]. In high salinity environments, a diffusional inflow of ions needs to be counteracted by excretion of Na^+^ and Cl^-^ from the extracellular fluids. Thus, the Na^+^/K^+^ ATPase is the main driving force in the bulk transport of sodium and chloride ions, both regarding absorption and secretion [15]. The net direction of the ions transported will be determined by the absence or presence of other ion-transporting proteins and ion channels, as well as by the selective leakiness of the epithelial tight junctions, especially for Na^+^. 

Na^+^/K^+^-ATPase is not only important in specific osmoregulatory tissues, but exists in virtually all cells where it plays an important role in maintaining the cell’s resting membrane potential, volume, and in providing a Na^+^-gradient for Na^+^-coupled transport. The NAK-generated Na^+^ gradient is, among other things, important in acid-base regulation including transport of protons [16]. In marine organisms in particular, the inwardly directed Na^+^ gradient would be beneficial for the excretion of an intracellular excess of protons by an apical membrane Na^+^/H^+^ exchanger [16]. Data on the importance of NAK in acid-base regulation come from several reports. NAK has been suggested to be involved in pH regulation in endosomes of vertebrate cells as well as in spermatozoa [17-19] and NAK expression has been shown to respond to changes in pH/pCO_2_ [20-22]. Consequently we hypothesize that the predicted near-future pCO_2_ increase [1], resulting in ocean acidification, might affect the functional importance of NAK in marine organisms. In addition to its ion pumping function, Na^+^/K^+^ ATPase is also involved in different signaling pathways via interaction with other proteins [23], in regulation of membrane trafficking [24], and in the functioning of adherens/tight junctions [25,26]. 

The main aim of this study was to increase our understanding of the molecular mechanisms behind the broad salinity tolerance of *B. improvisus*. We also investigated the possibility that near-future levels of ocean acidification might affect NAK expression in barnacles. The study was done by cloning and characterizing the *B. improvisus* Na^+^/K^+^ ATPase α-subunit and investigating its mRNA expression in different life stages, tissues, salinities and pCO_2_ levels. We found that the α-subunit of the Na^+^/K^+^ ATPase is encoded by two gene variants, *NAK1* and *NAK2*, where *NAK1* seems to exist as two very similar paralogs in the genome (*NAK1a* and *NAK1b*). Most importantly, *NAK1* is expressed as transcript variants encoding proteins with different length of their N-termini, being produced by alternative splicing. Interestingly, the long NAK1 mRNA form is up-regulated in relation to the short form during low salinity conditions, indicating the long Nak1 protein might have a more prominent functional role in low salinity osmoregulation. 

## Results

### Cloning of cDNA of the α-subunit of Na^+^/K^+^ ATPase 1 (Nak1) from B. *improvisus* revealed a variable N-terminus

**Figure 1:**
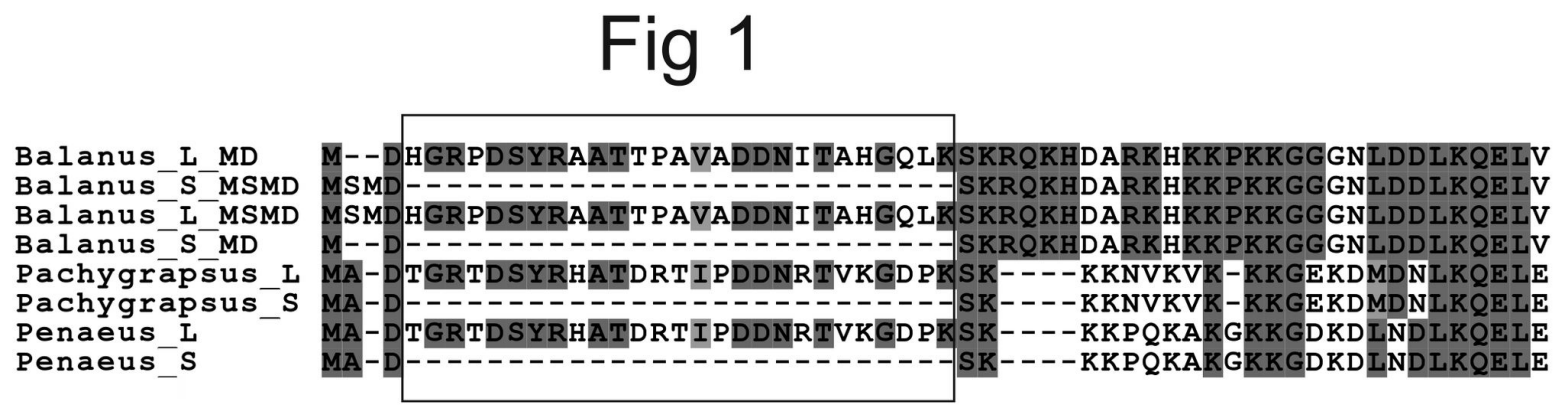
Alignment of *B. improvisus* Nak1 N-terminal variants to Na^+^/K^+^ ATPase sequences from the shrimp *Penaeus monodon* and the crab *Pachygrapus marmoratus*. Cloning of the *B. improvisus* Nak1 resulted in genes where the encoded proteins differed in length of their extreme N-termini. Comparison of the *B. improvisus* Nak1 N-termini with the N-termini of NAKs from two other crustaceans (*P. monodon* and *P. marmoratus*) revealed two main variants for each organism including or excluding a stretch of 27 amino acids. *B. improvisus* showed additional variability having either two (MD) or four (MSMD) amino acids before the 27 amino acid stretch, whereas the other two crustaceans had three amino acids (MAD). L and S stand for long and short form, respectively, and the box highlight the 27 amino acid stretch in the three organisms.

We have earlier obtained expressed sequenced tags (ESTs) from a batch of *B. improvisus* cyprid larvae from the Swedish west coast (Alm Rosenblad et al., unpublished data). In this sequence material we identified several ESTs for the α-subunit of a Na^+^/K^+^ ATPase, jointly covering roughly 60% of the central part of the encoded protein as judged by comparison with the full-length protein sequence from other crustaceans. To obtain the full-length cDNA sequence of the Na^+^/K^+^ ATPase from *B. improvisus*, rapid amplification of cDNA ends (RACE), was performed to identify the 5´ and 3´ ends, using RNA from cyprids as template. Interestingly, four different lengths of the coding part for the extreme N-terminus were found among the 5´ RACE products, where two of the obtained 5’ RACE clones contained nucleotides coding for a 27 amino acid sequence not present in the other two (Figure 1). In addition, the clones differed by having either two (MD) or four (MSMD) amino acids at the very N-terminus. No major variability was found in the rest of the coding part, other than occasional single nucleotide polymorphisms. Based on the obtained RACE sequences, primers were designed to PCR-amplify the whole open reading frame using cDNA from cyprids as template. We sequenced two PCR fragments that coded for two Na^+^/K^+^ ATPases of different lengths (1044 and 1017 amino acids). The two full-length clones were almost identical over the whole extent of the encoded protein, except in the extreme N-termini where one clone had the same 27 amino acid sequence insertion as found in some of the RACE products. Our sequences were compared with cloned cDNAs for Na^+^/K^+^ ATPase from the crab *Pachygrapsus marmoratus* [27] and the shrimp *Penaeus monodon* (EF672699, DQ399796) which also exhibit two N-terminal protein variants, one being 27 amino acids longer than the other (Figure 1). 

### The 27 amino acid N-terminal insertion is encoded by a separate exon in B. *improvisus*

**Figure 2:**
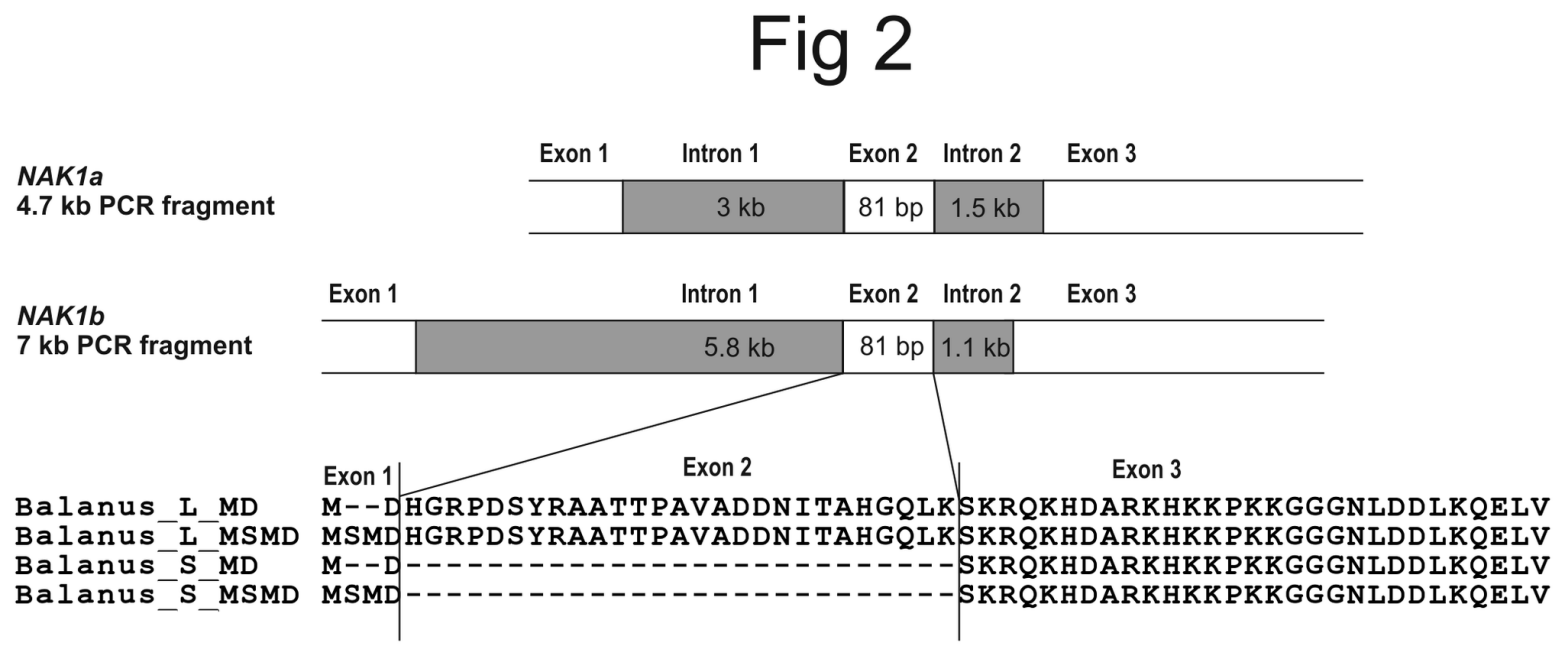
The 27 amino acid stretch of the longer N-terminus of Nak1 is encoded by a distinct exon. PCR on genomic DNA from a pool of cyprids was performed to investigate the genomic structure leading to the variable N-terminus of Nak1. Two different products (NAK1a and NAK1b) were obtained of about 4.7 and 7 kb, and revealed that the 81 nucleotide insertion, corresponding to the longer variants of the cDNA, is encoded by a separate exon (exon 2). In the Nak1a clones MD or MSMD were found at the predicted translation start, whereas only MSMD was found in the NAK1b clones. L and S stand for long and short form, respectively.

To investigate the genomic organization of the variable N-terminus of the Na^+^/K^+^ ATPase in *B. improvisus*, PCR was performed on genomic DNA from a pool of cyprid larvae. Forward primers based on sequences around the start ATG were combined with reverse primers just downstream of the 81 nucleotide fragment specific for the long protein variant (coding for the 27 amino acid N-terminal stretch). Two different PCR products of about 4.7 and 7 kb were obtained and named NAK1a and NAK1b, respectively. In both NAK1a and NAK1b the 27 amino acid N-terminal stretch was encoded by a separate exon (exon 2) (Figure 2). We found that the introns upstream of exon 2 were 3 and 5.8 kb and that the introns downstream were 1.5 and 1.1 kb for NAK1a and NAK1b, respectively. In addition, two variants of the exon containing the predicted translation start (exon 1) were found, coding for either two or four amino acids (MD or MSMD). Both variants were found in the NAK1a clones, whereas only MSMD was found in the NAK1b clones. The two variants at the translation start region were also seen in the cDNA 5´ RACE products (Figure 1). 

The different Na^+^/K^+^ ATPase sequence variants obtained using a pool of cyprid larvae could either be allelic variants of the same gene or be different genes. Therefore, in order to investigate the number of Na^+^/K^+^ ATPase genes in the *B. improvisus* genome, PCR amplification was conducted using genomic DNA from one single adult. Two separate PCR reactions were performed amplifying either the first or the second intron. Sequencing of thirteen clones containing the 3 kb long intron 1 of NAK1a resulted in two different sequences encoding either the amino acids MD or MSMD at the translational start. Sequencing of PCR clones containing intron 2 resulted in three different sequences of which two exhibited similarity to intron 2 in NAK1a and one showed similarity to intron 2 in NAK1b. Assuming that *B. improvisus* is diploid [28], our finding of three different Na^+^/K^+^ ATPase intron 2 sequences obtained from one single adult indicates that there are at least two Na^+^/K^+^ ATPase genes. We therefore assumed that we have partially cloned and sequenced two very similar genes with different intron 1 and intron 2 sizes, genes that we designate *NAK1a* and *NAK1b* (Figure 2). We believe that the two sequence variants at the N-terminus of clones with the 3 kb intron 1, MD and MSMD, are two different alleles of *NAK1a* based on the fact that the corresponding PCR fragments have very similar intron sequences. 

Comparison of the coding part of the 5’ end of the cDNA clones with the corresponding region of the genomic clones of *NAK1a* and *NAK1b* suggests that the long and short protein variants are the result of alternative splicing of the mRNA, which include or exclude exon 2 coding for 27 amino acids. However, to exclude the possibility that the short cDNA variant could originate from a gene lacking exon 2, PCR was performed using all pair-wise combinations of three different forward primers in exon 1 and two different reverse primers in exon 3 using genomic DNA as template. No PCR fragments bigger than 7 kb were obtained, and any fragments smaller than 4.7 kb (the fragment size expected for the *NAK1a* gene) turned out to be non-specific PCR products not at all related to Na^+^/K^+^ ATPases. We thus conclude from the PCR analyses that no gene lacking exon 2 appears to exist in the genome of *B. improvisus*. Consequently, our data suggest that the shorter variant of cDNA from the cyprid larvae originates from alternative splicing of the *NAK1a/b* mRNA. Furthermore, there are no differences in the splice sites surrounding exon 2 in *NAK1a* and *NAK1b*, making it unlikely that one of the genes is able to produce only the short variant.

### Cloning of Na^+^/K^+^ ATPase 2 (Nak2) from cDNA of B. *improvisus*

To investigate the possible existence of more Na^+^/K^+^ ATPase genes in addition to *NAK1a* and *NAK1b*, PCR was performed using degenerate primers complementary to a conserved region in the central part of arthropod Na^+^/K^+^ ATPases [29]. Genomic DNA from a batch of cyprid larvae was used as template and two bands of approximately 1,000 and 720 bp in size were obtained. Sequencing revealed that the 1,000 bp fragment represented part of the *NAK1* gene containing an intron. The other fragment (720bp), however, encoded a new Na^+^/K^+^ ATPase α-subunit with sequence similarity to Nak1 (see below), which we call Nak2. To obtain the complete protein sequence encoded by the *NAK2* gene, 3´ and 5´ RACE was performed separately on cDNA from two different adults. Based on the obtained RACE sequences, primers were designed to PCR-amplify the open reading frame. Two cDNA variants in each individual were found that were 96-97% identical and that we believe represent different alleles, coding for a protein of 1025 amino acids. 

### Next generation sequencing revealed the complete genomic structure of the Na^+^/K^+^ ATPase genes

As outlined above, the PCR amplification of the *NAK1* 5´ end from genomic DNA indicated that no *NAK1* gene lacking exon 2 exists in *B. improvisus*. However, we could not fully exclude the existence of a *NAK1* gene containing a very large intron between exon 1 and exon 3, resulting in a potential PCR fragment much bigger than 7 kb that would be hard to amplify and being outcompeted by the shorter fragments representing *NAK1a* and *NAK1b* (PCR fragment sizes of 7 and 4.7 kb, respectively). We therefore also searched for indications of a *NAK1* gene lacking exon 2 by examining sequences from our ongoing genome-sequencing project of *B. improvisus*. Contig assembly from 50 Gbp of Illumina reads from DNA of one single adult barnacle, yielding roughly 50-fold coverage, resulted in about 20 contigs covering the *NAK1* genes.

**Figure 3:**
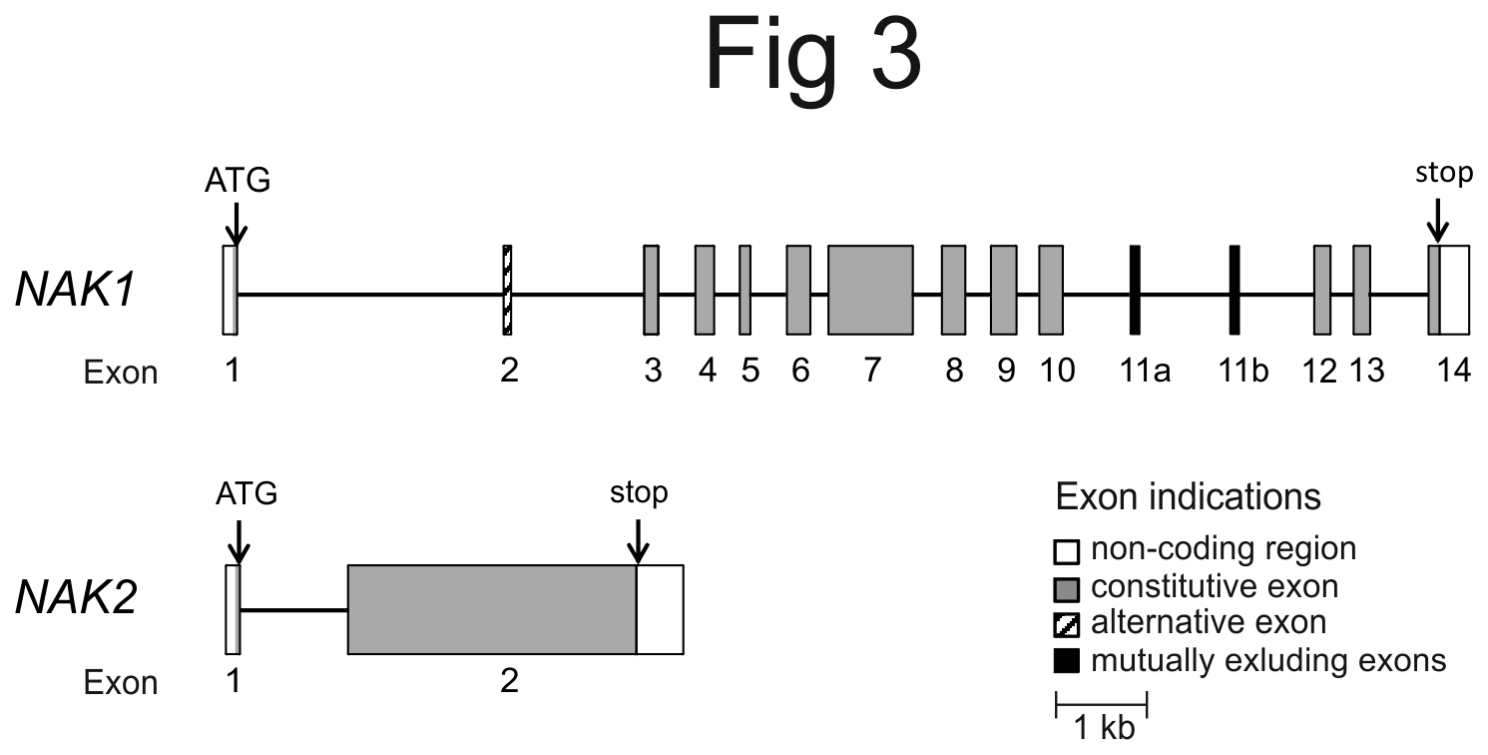
Genomic structure of *NAK1* and *NAK2.* PCR data together with data from Illumina sequencing were used to assemble the gene structure of *NAK1* and *NAK2*. *NAK1* contains 15 exons and 14 introns, whereas *NAK2* only has two exons.

We obtained good support in the Illumina sequence data for the exons and introns in the N-terminal part of *NAK1* that we found in the PCR studies. In addition, the full gene structure with the corresponding exon/intron break points could be assembled (Figure 3). We found that the gene structure of *NAK1* consists of 15 exons and 14 introns. The size of the gene is estimated to be 14-17 kb, with the first intron being relatively large (3 or 5.8 kb for *NAK1a* and *NAK1b*, respectively), followed by smaller introns of about 190-1500 bp in size. Exon sizes vary from 81 to 912 bp (Figure 3). Importantly, we found no contig where exon 1 and exon 3 were directly linked without any intron in between, or contigs where these two exons were separated by a longer and different type of intron. In addition, we obtained contigs representing four sequence variants covering exon 1 and parts of intron 1 in this single adult, further supporting the PCR results that indicated the existence of more than one *NAK1* gene. Two of the contigs were similar to the PCR clones of *NAK1a* and two were more similar to *NAK1b*. We thus conclude that both the PCR-based and genome sequencing-based search for *NAK1* variants lacking exon 2 were fruitless. Both methodologies showed the same exon/intron organization of *NAK1*, present at two different genomic loci (*NAK1a* and *NAK1b*), producing a short and long protein variant via alternative splicing. 

The Illumina sequencing showed the gene structure of *NAK2* to be very different from that of *NAK1* (Figure 3). The first amino acid of the protein (methionine) is encoded in a separate exon (exon 1) followed by an intron, and then the rest of the protein is encoded by only one large exon (exon 2). The intron was not completely covered in the Illumina sequences, but PCR on genomic DNA from two adults resulted in sequences containing an intron with an average size of about 1 kb.

### Sequence similarity and phylogenetic relationships of the cloned B. *improvisus* Na^+^/K^+^ ATPases

**Figure 4:**
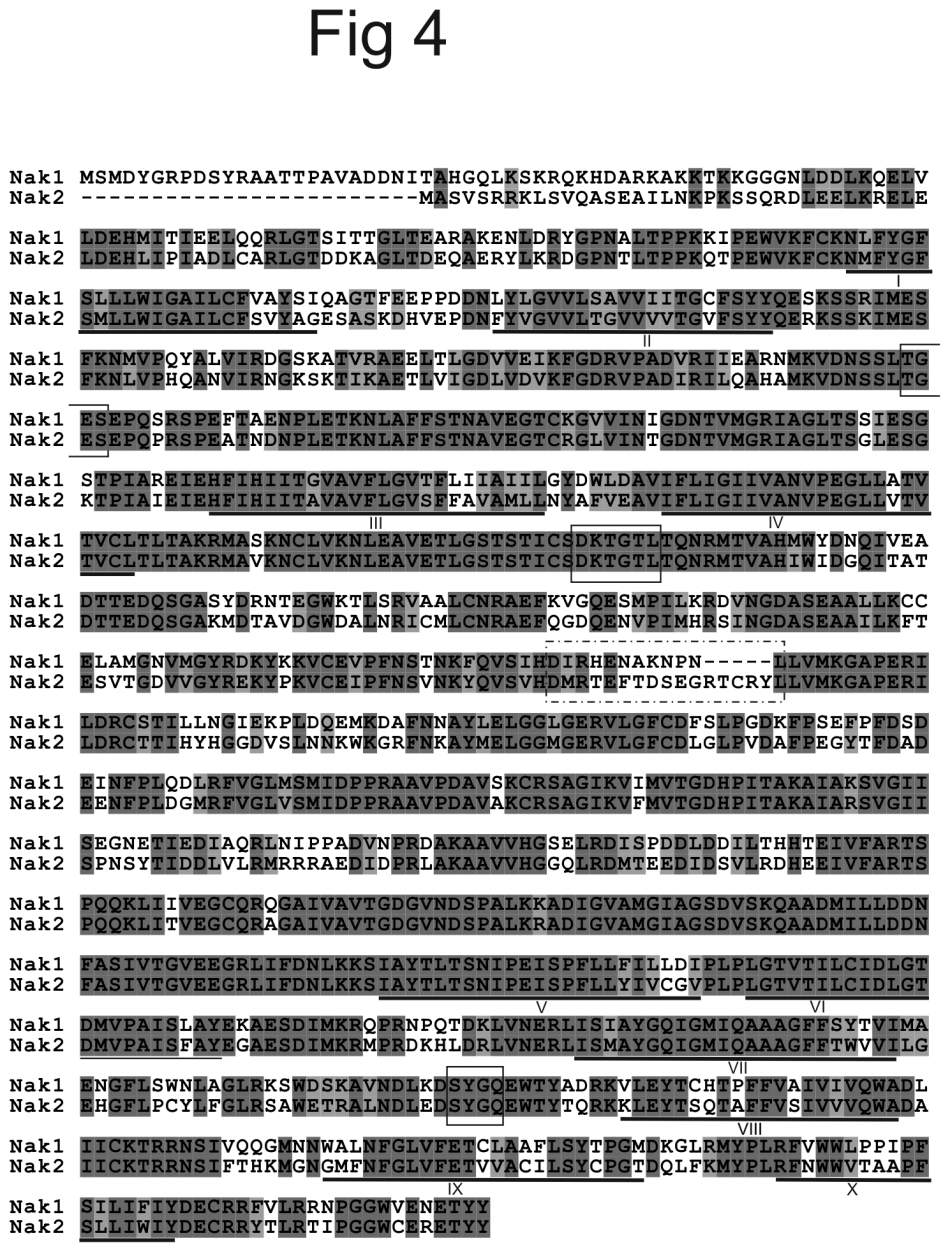
Protein alignment of the cloned Nak1 and Nak2. A protein alignment of the cloned Nak1 and Nak2 show that they are about 70% identical and that the largest sequence differences are in the N-terminus and in an area around amino acid 520. The isoform-specific region around amino acid 520 is marked with a dotted-line box and motifs conserved in NAKs are marked with solid-line boxes. Dark grey indicates identical amino acids and light grey functionally similar amino acids. Trans-membrane helices of Nak1, as predicted from TMHMM (http://www.cbs.dtu.dk/services/TMHMM/), are underlined and named with roman numbers.

Sequence comparison of Nak1 and Nak2 from *B. improvisus* showed that they were about 70% identical at the amino acid level. The most notable sequence differences were found in the N-termini and around amino acid 520 in Nak1. The region around amino acid 520 has earlier been shown to be variable between different isoforms of vertebrate Na^+^/K^+^ ATPases [30,31]. Here, Nak2 has a five amino acid insertion, RTCRY, compared to Nak1 (Fig. 4). Using cDNA libraries from larvae and adults of the related barnacle species *B. amphitrite* [32,33], two Na^+^/K^+^ ATPases were assembled, which were 96% and 94% identical at the protein level to the *B. improvisus* Nak1 and Nak2, respectively. The N-terminus and the isoform-specific regions around amino acid 520 of Nak1 and Nak2 were identical in *B. improvisus* and *B. amphitrite*. In addition, the long and short N-terminal variants of Nak1 in *B. improvisus* were found also in the *B. amphitrite* cDNA. However, in this case only variants with two amino acids (MD) before the identical 27 amino acid sequence were present. It should be noted that *B. amphitrite* NAK2 sequences were only found in the adult cDNA libraries.

Both Nak1 and Nak2 of *B. improvisus* contain conserved amino acid motifs previously shown to be important for enzyme activity of Na^+^/K^+^ ATPases in other organisms. These include a motif with the aspartate that is phosphorylated by ATP during the transport process (DKTGT(L/I/V/M)(T/I)), the motif SYGQ that has been shown to interact with the β-subunit of the ATPase complex, and the motif TGES in the A-domain involved in dephosphorylation of the aspartate in the catalytic cycle (Figure 4) [34,35]. Alignment with the recently crystallized and structure-determined shark Na^+^/K^+^ ATPase, showed that all amino acids in the K^+^ binding site in the *B. improvisus* protein are conserved as well as amino acids interacting with the FXYD proteins [36]. 

**Figure 5:**
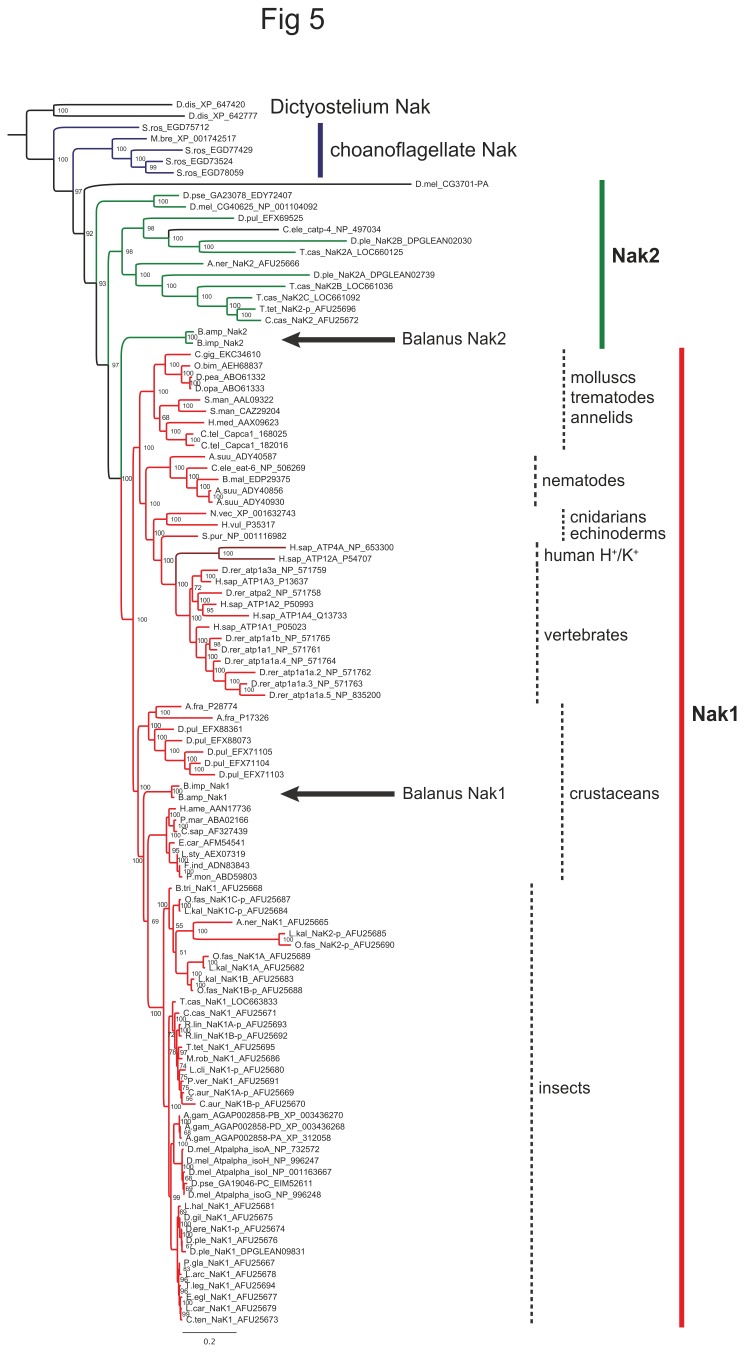
Phylogenetic relationships of the cloned *B. improvisus* NAKs. A phylogenetic tree was constructed using N-terminal truncated NAK sequences from *B. improvisus* and other metazoan organisms, e.g. humans, zebrafish, insects, crustaceans, and other invertebrates, as well as choanoflagellates. As outgroup, Na^+^/K^+^ ATPases from the slime mold *Dictyostelium* were used. In addition, human H^+^/K^+^ ATPase was added. The *B. improvisus* Nak1 sequence was found in the same clade as other arthropod Nak1 sequences. However, no clear-cut orthologues to the *Balanus* Nak2 were found. In general, Nak2 sequences have diverged more rapidly in relation to Nak1. Nak1 branches are shown in red, Nak2 branches in green. The divergent *Drosophila* NAK (CG3701) branch and the *C. elegans* catp-4 branch are shown in black. Choanoflagellate NAKs are shown in blue. *Balanus* Nak1 and Nak2 are marked with arrows. Scale bar shows expected number of changes per site. For abbreviations of species names and accession numbers see Table S3.

A phylogenetic analysis comparing Na^+^/K^+^ ATPase sequences from a broad range of metazoan organisms, showed that the *B. improvisus* Nak1 sequences were found in the same clade as other arthropod Nak1. In contrast, the *Balanus* Nak2 sequence and some other Nak homologs, were found on their own branches outside the metazoan NaK1 clade, thus preventing clear ortholog relationships to be established (Figure 5). 

### Differential expression of NAK1 and NAK2 mRNA is dependent on life stage and tissue type

**Figure 6:**
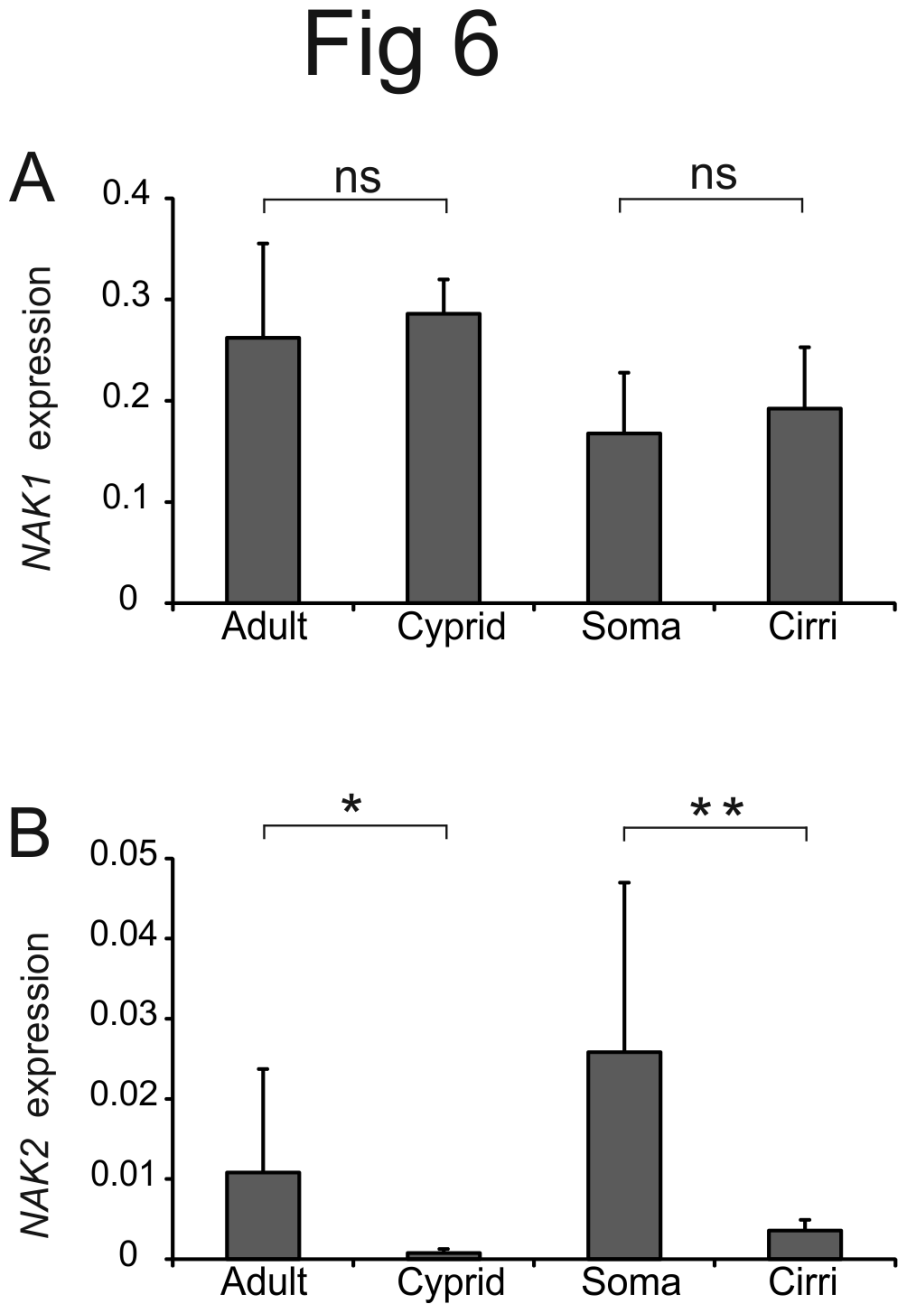
Differential expression of *NAK1* and *NAK2* in different life stages and tissues. QPCR was performed to compare the mRNA expression of the *NAK1* and *NAK2* genes between adults (n=10) and cyprids (n=5), and between the soma (n=14) and cirri (n=14) within an adult. For NAK1, primers detecting both the short and long N-terminal variants were used. The expression was normalized to the geometric average of five different reference genes. Error bars show the standard deviation. **A**) There was no significant change of *NAK1* expression between cyprids or adults (t-test, *P*=0.485) or between soma and cirri (paired t-test, *P*=0.206). **B**) Comparison of *NAK2* expression between cyprids and adults showed that NAK2 was very lowly expressed in cyprids compared to the adult barnacles (t-test, *P*=0.036). *NAK2* expression was also very low in the cirri compared to the soma in the adult barnacle (paired t-test, *P*=0.002). The same samples were used for analysis of both *NAK1* and *NAK2* expression.

To provide functional information on the *NAK1* and *NAK2* genes we wanted to link NAK expression to osmoregulatory capacity. We therefore initially investigated whether the *NAK1* and *NAK2* genes are differentially expressed in cyprids and adult barnacles as well as in two different tissues (soma and cirri) of the adult. The larvae of some crustaceans have been shown to be more sensitive to low salinities compared to adults [37-40] and barnacle tissues like cirri and the mantle epithelia have been suggested to be of importance for osmoregulation [9]. Expression of the Na^+^/K^+^ ATPases was normalized to the geometric average expression of five commonly used reference genes, actin, EF1 (elongation factor alpha 1), NADHd1 (NADH dehydrogenase subunit 1), RPL8 (ribosomal protein L8), and 36B4 (RPLP0, ribosomal protein P0), of which the three first have earlier been proposed as good references in barnacles [41]. The stability of the reference genes was evaluated using different methods (see Material and Methods, Figures S1-S3 and Table S4). There was no significant difference in *NAK1* expression between adults and cyprids (t-test, *P*=0.485) or between cirri and soma (paired t-test, *P*=0.206) (Figure 6A). In contrast, *NAK2* expression was significantly lower in cyprids compared to adult barnacles (t-test, *P*=0.036) with a roughly 14-fold difference in expression value (Figure 6B). We also found *NAK2* to be much lower in cirri compared to soma (~7-fold) in the adult (paired t-test, *P*=0.002) (Figure 6B). 

**Figure 7:**
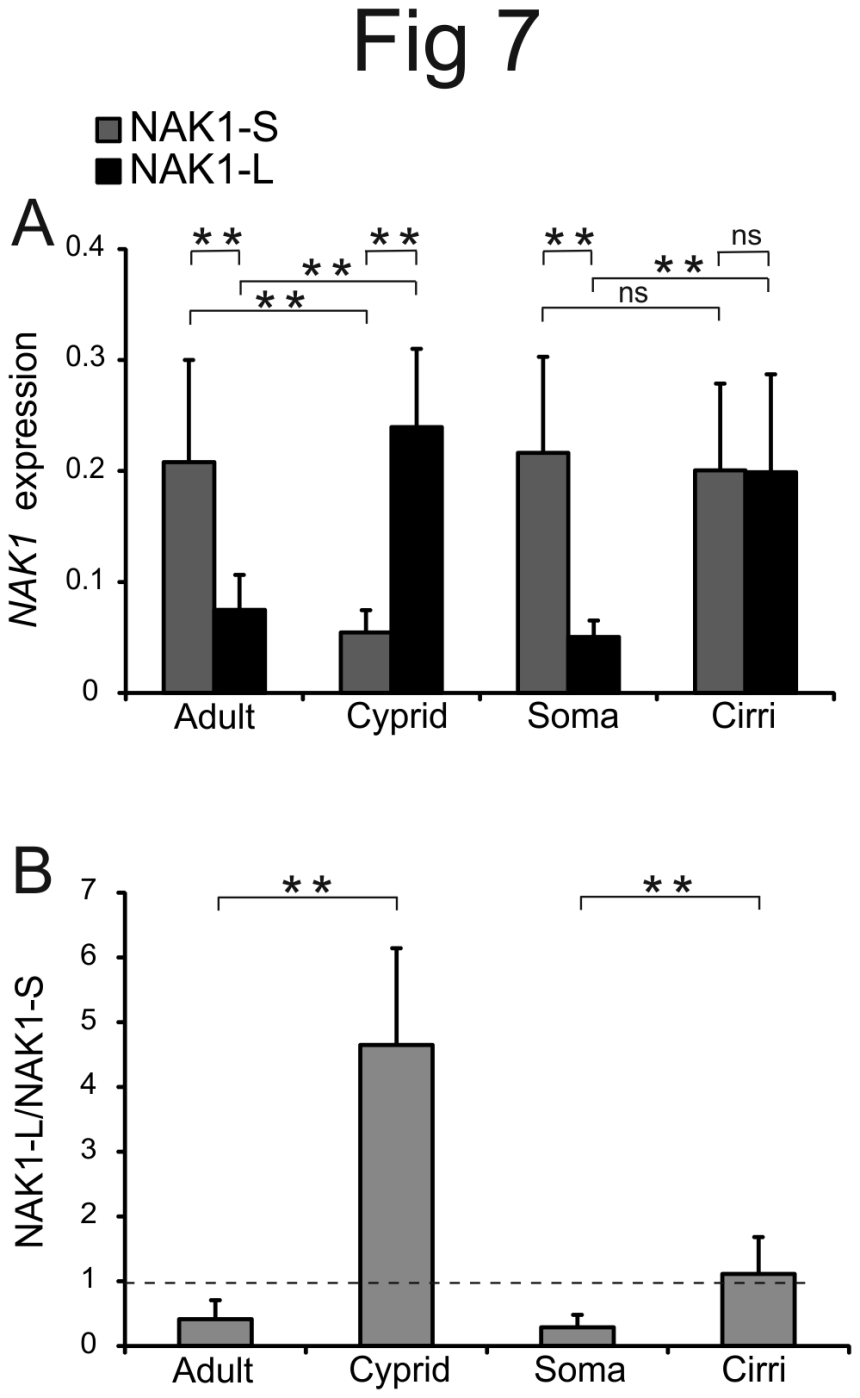
Differential expression of the two splice variants of *NAK1* in different life stages and tissues. QPCR was performed to compare the mRNA expression of the short (NAK1-S) and long (NAK1-L) NAK1 isoforms between adults (n=10) and cyprids (n=5), and between the soma (n=14) and cirri (n=14) within an adult. Error bars show the standard deviation. **A**) Cyprids express more of the long than of the short isoform (paired t-test, *P*=0.002), whereas the short isoform is dominating in adult individuals (paired t-test, *P*=0.001). In the cirri of adults there is about equal expression of the long and short isoform (paired t-test, *P*=0.959), whereas in the soma the short isoform is dominating (paired t-test, *P*<0.001). The expression was normalized to the geometric average of five different reference genes. **B**). The relative expression of the long variant compared to short variant is significantly higher in cyprids than in adults (t-test, *P*=0.003) and in cirri compared to soma (paired t-test, *P*<0.001).

Next we searched for expression differences of the N-terminal splice variants of *NAK1* using qPCR primers specific for the long and short isoforms. However, mRNA from *NAK1a* and *NAK1b* could not be distinguished because of the great sequence similarity in their coding parts. It should be noted that the genomic sequence of *NAK1b* has some nucleotide changes in exon 1 and exon 3 compared to *NAK1a*. We did not find any of these *NAK1b* specific sequence variants in the cDNA clones obtained from cyprids, indicating that gene *NAK1b* might be expressed at a low level or not at all in that life stage. It was also not possible to make primers selecting between the two mRNA variants encoding proteins only differing by two amino acids in the N-terminus (variants containing the MD or MSMD). Comparing different life stages, we found a roughly 4-fold dominance of the longer *NAK1* mRNA (that encodes the 27 amino acid N-terminal part) over the short variant in cyprids (paired t-test, *P*=0.002), whereas in the adult, the short isoform was clearly dominant (paired t-test, *P*=0.001) displaying about 3-fold higher expression (Figures 7A, B). Cirri expressed approximately four times more of the long variant than the soma (*paired* t-test, P<0.001), whereas there was approximately equal expression of the short variant in the two tissues (paired t-test, *P*=0.617) (Figure 7A). We conclude that there are both life stage and tissue specific expression differences for the two isoforms encoded by *NAK1*, with the long NAK1 being the predominant variant in cyprids and cirri. 

### The long NAK1 mRNA containing exon 2 is predominant at low salinities

**Figure 8:**
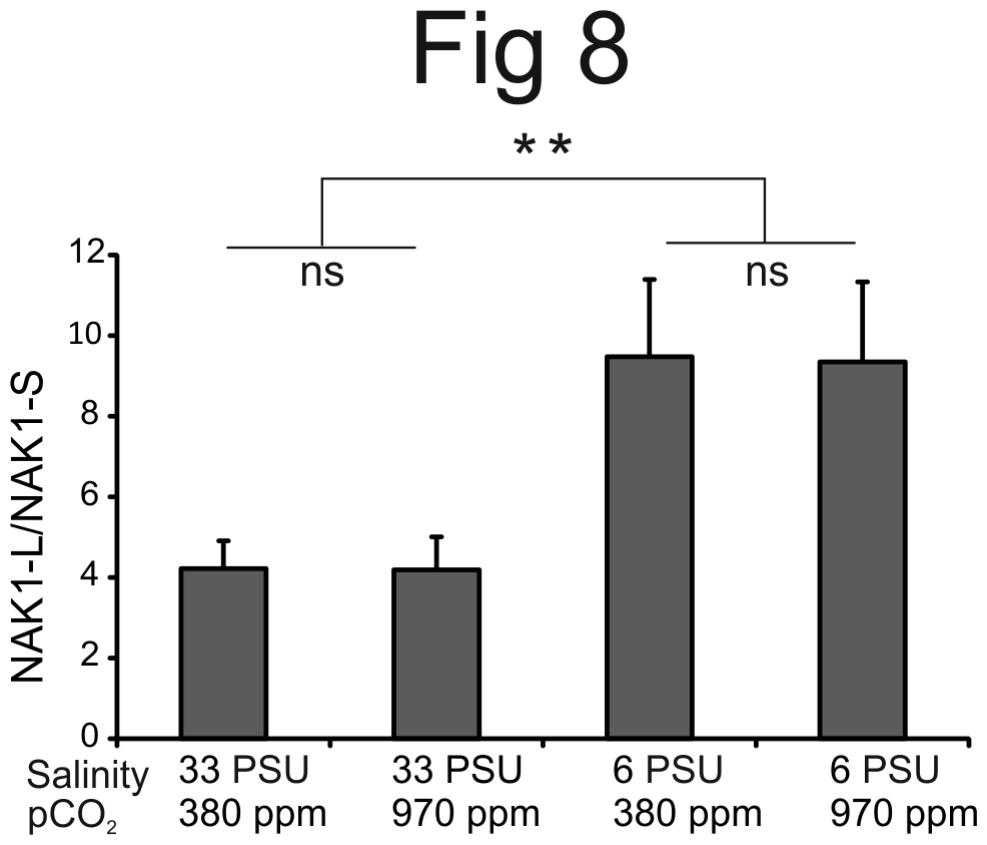
Differential expression of the splice variants of *NAK1* in response to various salinities and pCO_2_ levels. QPCR was performed to measure the mRNA expression of the short (NAK1-S) and long (NAK1-L) NAK1 isoforms in a cyprid population exposed for 24 hours to combinations of salinities of 33 PSU or 6 PSU and pCO_2_ levels of 970 or 380 ppm. The experiment was performed on four different batches of cyprids for each treatment. Error bars show the standard deviation of the four repeats. In low salinity treatment the expression of the long isoform is increased relative to the short (ANOVA, *P*<0.001).

To further characterize the functional role of NAK in *B. improvisus*, we investigated whether the NAK expression, in particular the *NAK1* splice variants, was responsive to changes in two important environmental cues; salinity and pCO_2_. Cyprids from marine conditions were exposed to combinations of two different salinities (33 PSU or 6 PSU) and two different pCO_2_ levels (970 or 380 ppm) for 24 hours. The higher pCO_2_ level was chosen to reflect future climate scenarios for the year 2100 [1]. These treatments were part of a larger study on effects of multiple stressors on barnacle gene expression patterns (Wrange et al. unpublished). We found that all our five control genes consistently exhibited slightly lower expression in the low salinity treatment (Figure S4). Since the same amount of total RNA was used for all samples in the qPCR analysis, either these control genes were all down-regulated during the low salinity treatment or the relative content of mRNA in the total RNA pool decreased. In either case, the apparent change in expression in the low salinity treatments made normalization to these reference genes uncertain and was therefore not performed. However, comparison of the relative expression of the long and short variants between the different treatments remains valid. Relative expression of the *NAK1* long mRNA increased about 2-fold in low salinity (ANOVA, *P*<0.001) (Figure 8; for average Ct values see Figure S5A). In contrast, no significant effect of pCO_2_ was seen on expression of the *NAK1* isoforms in neither high nor low salinity (Figure 8). There were also no major changes in total expression of *NAK1* (ANOVA, *P*=0.708) or of *NAK2* expression (ANOVA, *P*=0.072) between treatments (Figures S5B, C). Minor expression changes would be hard to detect since the expression data were not normalized and since expression of *NAK2* in cyprids was very low. Collectively, our data indicate that the alternative splicing of the *NAK1* genes is controlled by life stage, tissue type and environmental salinity, with the long form being predominant in cyprids, cirri of adults and under low salinity conditions. 

## Discussion

### Multiple isoforms of Na^+^/K^+^ ATPase in the barnacle B. *improvisus*

Here we present the first molecular characterization of a Na^+^/K^+^ ATPase α-subunit from barnacles. We isolated the gene from the euryhaline species *B. improvisus*, which exhibits an unusual tolerance to low salinities compared to almost all other barnacles [3,42]. Indeed, *B. improvisus* shows maximum performance at low and intermediate salinities and can be regarded as one of few truly brackish-water species [43,44]. In other crustaceans, Na^+^/K^+^ ATPase appears to be of importance for the ability to survive, grow and reproduce in both high and low salinities [6]. Therefore, this ion transporter is of interest for understanding the physiology and ecology of *B. improvisus*, in particular in relation to its capacity for living in low salinity environments. 

We identified two main gene variants of the Na^+^/K^+^ ATPase, which we name *NAK1* and *NAK2*. They display different expression profiles in different tissue types and life stages, indicating that they may serve different functions. The encoded Nak1 and Nak2 proteins are 70% identical with the greatest sequence divergence between the two paralogs in the very N-terminal part where the Nak2 protein is completely unrelated to Nak1 (Figure 4). More than one copy of the Na^+^/K^+^ ATPase gene has also been reported in other crustaceans; six Na^+^/K^+^ ATPase genes/proteins have been annotated in *Daphnia pulex* (EFX88361.1, EFX88073.1, EFX71105.1, EFX71104.1, EFX71103.1 EFX69525.1) and two in *Artemia franciscana* (P28774.1, P17326.1) [45,46]. It should be stressed that *Artemia* and *Daphnia* (five of its six NAK genes) encode NAK-variants having N-termini with no similarity to the *Balanus* Nak1 or Nak2 proteins. Many insects also have multiple copies of NAKs that are divided into two classes; *NAK1* and *NAK2* [47]. Within both these classes, several recent duplications seem to have occurred resulting in multiple gene copies. Also in *B. improvisus* we found two very similar copies of *NAK1* (*NAK1a* and *NAK1b*), indicating a recent duplication. Our phylogenetic analysis reveals that the *B. improvisus* Nak1 was found in the same clade as most other arthropod Na^+^/K^+^ ATPase 1 sequences. However, the evolutionary relationships between the NAK homologs that do not belong to the Nak1 group are less clear. Thus, no definitive orthologs for the *Balanus* Nak2 could be identified. Although the support for the phylogenetic positions of the Nak2 clades was lower than for Nak1, we can conclude that the Nak2s are generally found on longer branches and are thus faster evolving than Nak1s (Figure 5). However, because of the scarcity of Nak2 data from several metazoan lineages, and that several duplications have occurred during evolution, it is presently not possible to establish the exact orthologous relationships between the Nak2 sequences. Overall we can conclude that that duplication of an ancestral NAK clearly occurred before the appearance of arthropods and furthermore that the existence of multiple NAKs in choanoflagellates indicates that some Nak2s may have an ancient origin [48]. 

Adding to the complexity of the NAK gene repertoire, we found that *B. improvisus* has two mRNA variants coding for Nak1 proteins of differing lengths. We show that the short and long variants of Nak1 are formed by alternative splicing of exon 2 (81 nt in length) that encodes a 27 N-terminal amino acid stretch. NAK protein sequences from several other crustaceans show similar N-termini containing variants of this conserved 27 amino acid sequence, e.g. the waterflea *D. pulex*, the shrimps *Fenneropenaeus indicus*, *Litopenaeus vannamei*, *Penaeus chinensis* and *P. monodon*, the lobster *Homarus americanus* and the crabs *Callinectes sapidus* and *P. marmoratus* (for accession numbers see Table S3). However, in crustaceans, mRNA transcripts of both the short and long Nak1 variant have only been reported for *P. marmoratus* [27] and *P. monodon* (EF672699, DQ399796). Whereas the NAK proteins from *B. improvisus* have either two (MD) or four (MSMD) amino acids preceding the 27 amino acid stretch, *P. marmoratus* and *P. monodon* have three amino acids (MAD) (Figure 1). Furthermore, the 27 amino acid N-terminal stretch is completely conserved between *P. marmoratus* and *P. monodon*, whereas in *B. improvisus* only 13 amino acids are identical to the two decapod crustaceans. Insects also have Na^+^/K^+^ ATPase genes containing the 27 amino acid sequence and their gene structures reveal a similar organization to *B. improvisus* with a separate exon coding for the N-terminal 27 amino acid stretch. In the honeybee *Apis mellifera*, there are ESTs supporting the presence of transcripts with and without the 27 amino acid exon (DB741303, DB734954), which is similar to the situation in *B. improvisus*. In *Drosophila melanogaster*, however, the 27 amino acid exon is not alternatively spliced and in this species the transcripts lacking the 27 amino acid encoding region instead have a different transcriptional start site [49]. 

In addition to alternative splicing of the N-terminus, there seems to be possibilities for alternative splicing also further downstream in the *B. improvisus NAK1* gene: exon 11 existed in two tandem copies with highly similar protein sequences, denoted 11a and 11b (Figure 3). We have so far only detected transcripts with exon 11b in our cDNA sequences from cyprids, indicating that these exons (11a and 11b) are mutually exclusive in *B. improvisus* and that exon 11b is the preferred variant under the environmental conditions that we have examined. The genomic structure of *NAK* genes in *D. pulex* and several other arthropods show similar organization, having two to four copies of the corresponding exon 11 in the gene (*Daphnia* JGI_V11_305634, *Anopheles* AGAP002858, *Ixodes* ISCW002538). In *Drosophila*, alternative spliced transcripts containing either of the four similar, but not identical, exon 11 copies were found, indicating that these exons also are mutually exclusive [49]. The functional importance of exon 11a and 11b in the encoded protein variants needs to be elucidated.

### Gene organization of crustacean Na^+^/K^+^ ATPase N-termini

The gene organization of the N-terminus of the Na^+^/K^+^ ATPase gene in *P. marmoratus* and *P. monodon*, resulting in the long and short transcripts, appears to be different to that of *B. improvisus* and insects. In the case of *P. marmoratus* only the shorter variant lacking the 27 amino acid sequence was found when running PCR on genomic DNA [27]. In *P. monodon*, a genomic sequence (FJ360440) showed that the conserved 27 amino acid sequence was not encoded for by a separate exon, but part of a larger exon. Thus, in *P. monodon* the sequences corresponding to exon 1, 2 and 3 in *B. improvisus* are fused without the interruption of introns, and the short NAK variant must therefore be encoded for by a different gene. Since, at the genomic level, only the short variant was found in *P. marmoratus* and only the long variant without splice possibilities in *P. monodon*, these organisms might each have two, very similar genes, one expressing the short and the other the long variant. In *B. improvisus*, we did not find any *NAK1* gene lacking the 81-nucleotide exon, despite extensive efforts including PCR on genomic DNA with different primers as well as next generation sequencing of the genome with good coverage. It should be stressed that we found two genes for *NAK1* (*NAK1a/NAK1b*); however, both genes contained exon 2. Thus, in *B. improvisus*, we suggest that the N-terminal variants are created by alternative splicing of the *NAK1a/b* genes resulting in insertion or exclusion of exon 2 coding for 27 amino acids. 

### The alternatively spliced forms of B. *improvisus* Na^+^/K^+^ ATPases Nak1 are differentially expressed

The relative expression of the *NAK1* isoforms was clearly differentially regulated during development. The long variant with the 27 amino acid N-terminal extension was the dominant isoform in cyprids, whereas the opposite was found in adults with higher expression of the short variant (Figure 7). The long Nak1 variant might be more important for osmoregulation in cyprids than in adults or could potentially be involved in processes especially important for cyprids, e.g. the functioning of the nervous system, which is more developed in cyprids compared to adults [50,51]. 

The main osmoregulating organ in adult barnacles is not known, but tissues like cirri and mantle epithelia have previously been suggested to be of importance in this process [9]. Our qPCR data (Figure 7A) showed that there was higher expression of the long NAK1 variant in cirri compared to soma, which could indicate a more important role of this long isoform in osmoregulation. Further support for an osmoregulatory role of the long form stems from the increased expression of the long variant relative to the short variant, when we subjected cyprids to low salinity. In a salinity experiment testing effects of hypo- and hyper-osmotic stress on the crab *Pachygrapsus*, the authors reported that both the short and long mRNA variants increased in gills, however, no analysis of their expression ratio was reported [27]. 

There are several examples of genes with differentially expressed splice variants with potential functional consequences in invertebrates, especially in *Drosophila*. One example is the troponin T gene (TnT) that has two tandem copies of exon 10 (10a and 10b) of which the isoform containing exon 10a is expressed specifically in indirect flight muscles and jump muscles [52,53]. The functional difference between isoforms containing either exon 10a or exon 10b is not known, but exon 10a has more potential phosphorylation sites [53]. Furthermore, in muscles of dragonflies, TnT splice variants are differentially correlated to Ca^2+^ sensitivity and force production [54]. Another example is the carnitine palmitoyltransferase (CPT) gene involved in lipid metabolism that has alternatively spliced transcripts containing either exon 6a or 6b that are differentially expressed in the flight muscle and fat body, and which have been shown to possess different kinetic properties [55]. Similar to these differentially expressed splice variants in insects, the different proteins encoded by the long and short splice variants of *NAK1* in *B. improvisus* might have different functions. However, their physiological role in different salinities needs further investigation. 

In addition to differential expression of splice variants, different *NAK* genes might respond differently to changing salinities. In *Artemia* only one of the Na^+^/K^+^ ATPase genes was up-regulated during salinity stress [56], and in euryhaline fish species, such as *Salmo salar*, different Na^+^/K^+^ ATPase isoforms are differentially expressed in response to environmental salinities [7,12,13]. In *B. improvisus* cyprid larvae, however, no apparent change in expression in relation to alteration in salinity was seen for total expression of NAK1 or NAK2 mRNA (Figures S5B, C). Although many other crustaceans show an increase in NAK expression and activity when exposed to both high and low salinities [14], there are also cases where no expression or activity changes occur. In such cases the organisms might conform or increase the expression of other ion-transporters [57,58].

There was no effect of pCO_2_ on the relative expression of the long and short *Balanus* NAK1 mRNA. A similar result was found in the crab *Carcinus maenas* where no change in expression of NAK was seen in response to decreased pCO_2_ [59]. In some other organisms, however, NAK expression or activity has been shown to be pCO_2_/pH dependent [20-22]. The connection between pCO_2_/pH and NAK expression and function is not well studied, but there are models connecting the NAK-generated Na^+^ gradient to acid-base regulation and NAK has been suggested to regulate pH in endosomes in vertebrate cells and in rat spermatozoa [16-19]. In addition, pH could potentially also directly affect NAK functionality as has been shown for its selectivity for K^+^ over Na^+^ at different pHs [60]. Under the latter scenario a change in NAK expression could be a reflection of compensation for altered functionality, however, we could not find any support for this in our NAK expression data. 

### Functional implications of the variable N-termini of Na^+^/K^+^ ATPase 1

**Figure 9:**
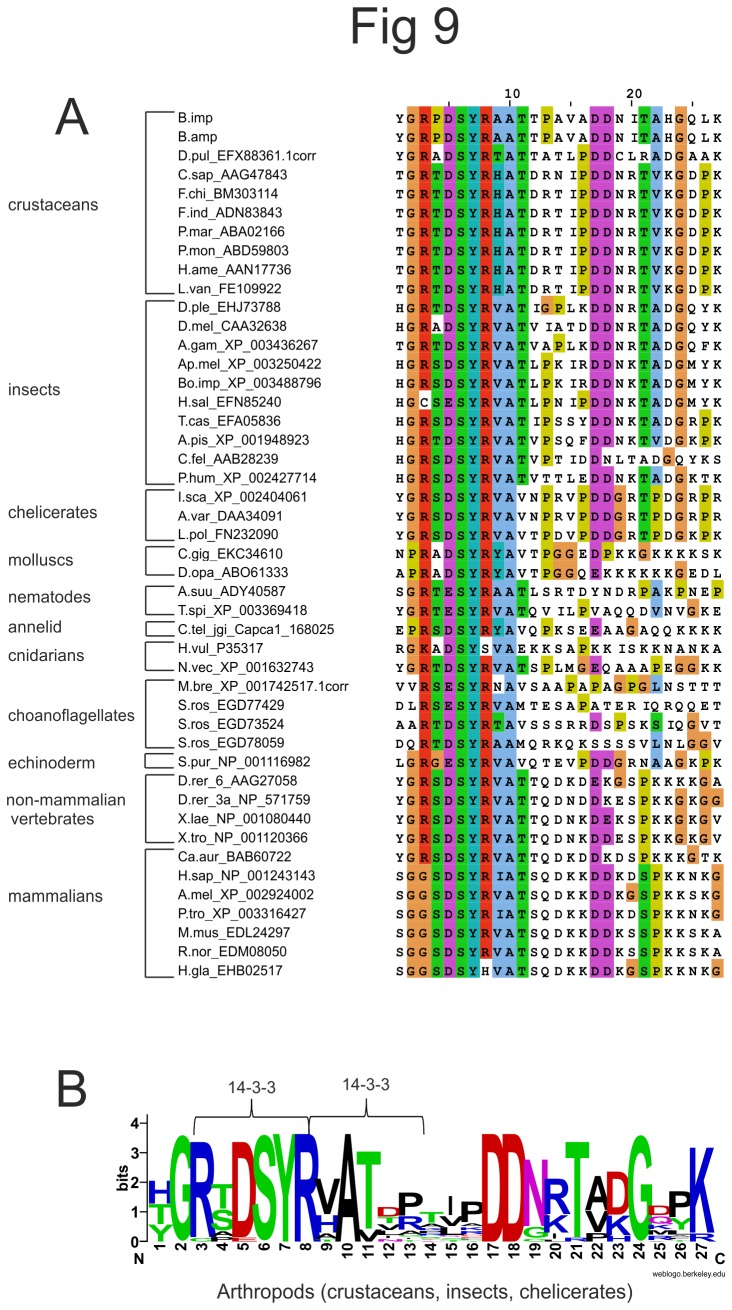
Conservation over wide evolutionary distances of potential 14-3-3 binding sites in the N-terminus of NAKs. **A**) An alignment of the conserved 27 amino acid sequence in the N-termini of NAKs from a wide range of organisms is shown. **B**) A sequence logo (http://weblogo.berkeley.edu) of the conserved 27 amino acid sequence from arthropods is shown and potential 14-3-3 binding sites are marked with brackets. For abbreviations of species names and accession numbers see Table S3.

Despite the fact that the N-terminus is the least conserved part of Na^+^/K^+^ ATPases, the 27 amino acid stretch encoded by the alternatively spliced exon 2 of *NAK1* in *B. improvisus* is partly conserved in Na^+^/K^+^ ATPases of a wide range of organisms, not only in crustaceans (Figure 9A). Most strikingly, this is the case even in the closest relatives to metazoans, choanoflagellates, indicating that the functional importance of this sequence motif is conserved over very large evolutionary distances. Among arthropods we found one strongly conserved motif GRxDSYRxAT (Figure 9B). This motif includes the earlier reported sequence (RTDSYR) in the N-terminus of the *P. marmoratus* Na^+^/K^+^ ATPase, where the phosphorylated serine is proposed to be the site for binding of 14-3-3 proteins [27]. The RTDSYR sequence also fulfills the criteria for the 14-3-3 binding motif Rxx**S/T**xR/P (bold indicates the phosphorylated residue) recently identified in an experimental study on 14-3-3 binding sites [61]. The motif occurs in the form of RxD**S**YR in *B. improvisus* as well as in almost all other arthropods. Interestingly, *B. improvisus* and most other crustaceans also have a potential second closely situated 14-3-3 binding site present as RxA**T**xR/P, starting with the last R in the first site (Figure 9B). In addition, there is experimental support for a physical interaction between 14-3-3 and the rat NAKα1, however, this NAK has an N-terminus lacking the 27 amino acid stretch [62]. 

14-3-3 proteins play important roles in several aspects of cellular physiology, including cellular transport, gene expression and cell survival [63]. To exert their action, 14-3-3 dimers dock onto phosphorylated sites on target proteins to influence their conformation and interactions to other proteins. In that way, 14-3-3 dimers can integrate multiple signal inputs to generate an effect, i.e. act as “digital logic gates” [64]. The 14-3-3 proteins have been shown to be important in salinity adaptation of plants, where it activates H^+^ ATPases [65,66]. There is also experimental evidence for the involvement of 14-3-3 protein in salinity tolerance in aquatic animals, e.g. in the fish *Fundulus heteroclitus* and in the shrimp *P. monodon*, where one isoform of 14-3-3 was up-regulated in gills during salt stress [67,68]. In addition, individuals of *P. monodon*, naturally lacking the osmotic stress induced isoform, were more sensitive to salinity stress [67]. Finally, a mechanistic link between 14-3-3 and NAK was indicated since shrimps treated with RNAi targeting the 14-3-3 transcript had less total ATPase activity [67]. We conclude that several different pieces of evidence indicate that 14-3-3 might play a role in salinity adaptation of crustaceans, possibly via interaction with the Nak1 protein. In this respect the increased relative expression of the long Nak1 isoform in barnacle larvae in low salinity is interesting and suggests that regulation of Nak1 activity, via 14-3-3 binding to the long Nak1 isoform, is of greater importance during low salinity conditions. 

In vertebrates, several proteins have been shown to interact with the N-terminus of NAK as part of signaling pathways. For example, hormone-stimulated endocytosis of the rat NAK has been shown to be dependent on the binding of phosphoinositide-3 (PI3) kinase and 14-3-3 to the N-terminus [62,69], and the inositoltriphosphate (IP3) receptor and caveolin1 have been shown to interact with the N-terminus when NAK functions as a signal transducer [70,71]. Similarly, the long Nak1 variant in barnacles might make protein interactions via the 27 amino acid stretch, and in that way be involved in different signaling pathways than the short variant. In addition, the N-terminus might influence the Na^+^/K^+^ transport function of NAK intramolecularly, since deletions of the N-terminus of the rat NAKα1 have been shown to affect conformation-changes that take place during the catalytic cycle [72]. Likewise, the long and short Nak1 forms in *B. improvisus* might affect the catalytic cycle in different ways.

In conclusion, we have identified various genes encoding the Na^+^/K^+^ ATPases in the euryhaline barnacle *B. improvisus*. We report that the mRNA of *NAK1* exists in variants with different length, the corresponding N-termini of the encoded protein being produced by alternative splicing leading to inclusion/exclusion of an exon encoding a 27 amino acid region. Most importantly from an osmoregulatory perspective, the expression of the long NAK1 mRNA increased relatively to the short isoform when barnacle cyprid larvae were subjected to low salinity, indicating that the long Nak1 protein might have a more prominent functional role in low salinity osmoregulation. Our findings provide an interesting basis for further investigation of the functional roles of the different *B. improvisus* NAKs in barnacle osmoregulation.

## Materials and Methods

### Culturing of B. *improvisus* including the rearing of cyprid larvae

The establishment of laboratory cultures of *B. improvisus* and the rearing of cyprids were as earlier described [73,74]. In short, cyprid larvae of *B. improvisus* were allowed to settle and metamorphose on plexiglass panels placed in the sea off the west coast of Sweden during June to August, in the vicinity of Tjärnö Marine Biological Laboratory (58° 53’N, 11° 08’E), Sven Lovén Centre for Marine Sciences at the University of Gothenburg. The panels with adults were then brought to the laboratory and placed in aquaria with running seawater and used as broodstock for cyprid larvae. Adults were taken from these laboratory panels for further analysis. The adult animals were removed from their shell by opening the operculum plates and pulling the animal out with forceps, excluding mantle or ovary tissue. For some QPCR studies, the cirri were detached from the soma (which we here define as the remaining part of the body after excision of the cirri).

### Nucleic acid preparation and cDNA synthesis

RNA or genomic DNA was prepared from the barnacles using the Qiagen RNeasy mini-kit (Qiagen) and the E.Z.N.A Blood DNA Kit (Omega Bio-Tek), respectively, as described in Lind et al. [73], with the modifications that adults were homogenized with a plastic piston in an eppendorf tube before sonication. RNA was used as template for cDNA synthesis using the SuperscriptIII first strand kit (Invitrogen). The genomic DNA and cDNA were used for cloning of NaKs and for mRNA expression analysis using real-time PCR.

### Cloning of Na^+^/K^+^ ATPases

To clone the NAK1 cDNA, 5´ and 3´ rapid amplification of cDNA ends (RACE) using the GeneRacerTM kit (Invitrogen) was performed with PCR primers based on ESTs from a previously sequenced library of *B .improvisus* cyprids (Alm Rosenblad et al. unpublished data). The sequences of the 5´ and 3´ RACE products were then used to design primers enabling cloning of the full-length protein. In order to clone the NAK2 cDNA, PCR with degenerate primers complementary to a conserved part of arthropod NAKs [29] was performed on genomic DNA prepared from a batch of cyprids. The sequence of the obtained fragment was used to design primers for use in 5´ and 3´ RACE. The products from the 5´ and 3´ RACE were then used to design primers enabling cloning of the full-length protein. Cloning of the Nak1 N-terminus from genomic DNA to investigate the intron/exon structure, was performed with PCR using primers based on sequences from the 5´ end of the cloned cDNA (see Appendix S1 as well as Table S1 and S2 for a more complete description of PCR procedures and primer sequences used).

### Quantitative Real-time PCR

Quantitative Real-time PCR (qPCR) was performed to compare the mRNA expression of the short and long isoform of *NAK1* and of *NAK1* and *NAK2*. PCR reactions containing iQ SYBR Green supermix (Bio-Rad), cDNA and primers specific for the different NAKs or for the reference genes, were run on an iQ5 iCycler (Bio-Rad). Primer efficiencies were determined from standard curves obtained by running qPCR on different amounts of cyprid cDNA. The obtained Ct values (cycle threshold values) were plotted against the logarithm of the cDNA concentration and the primer efficiency (E) was determined from the slope of the curve according to formula E=10^(-1/slope)^. Primer efficiencies for all examined genes were in the range of 92 to 110%.

### Evaluation of Quantitative Real-time PCR reference genes

In order to use qPCR for comparison of the mRNA expression of *Balanus NAKs*, an evaluation of the five commonly used qPCR reference candidate genes; actin, RPL8, 36B4, EF1 and NADHd1, was first performed. For reference gene primer sequences, see Table S1. For the analysis of expression in tissues and life-stages, in total 43 different samples were used consisting of soma and cirri separately from 14 adult individuals, 10 adults (soma connected with cirri, excluding mantle or ovary tissue) and 5 cyprid batches (~1000 individuals per batch). After running qPCR with primers for the 5 different reference genes on the 43 samples, we could conclude that there were no larger differences in expression levels of the respective reference gene between the sample groups; soma, cirri, adult or cyprid (Figures S1 and S2). The reference gene qPCR data were also analyzed with BestKeeper, NormFinder and geNorm [75-77]. These programs use different algorithms for calculating the stability of the reference genes. In addition geNorm also suggest the optimum combination and number of reference genes to be used as normalization factor. The three programs all ranked actin, EF1 and RPL8 as most stable and NADHd1 and 36B4 as a little less stable (Table S4). However, including all five reference genes for normalization was slightly better than using only the three most stable, according to geNorm (Figure S3). We therefore used the geometric average [77] of the expression of all five genes as a normalization factor (NF). Normalized NAK expression was calculated as NF/E^Ct(Nak)^. For comparison of reference gene expression in cyprids treated with different salinities and pCO_2_ levels, four cyprid batches for each of four treatments were used (in total 16 samples). The samples used for evaluation of the reference genes were also used for comparison of NAK expression.

### Exposure of cyprids to different salinities and pCO_2_ levels

To investigate the transcriptional response of *NAK*1 and *NAK*2 to different environmental cues, cyprid larvae were subjected to four treatments consisting of two salinities (33 PSU or 6 PSU) combined with two pCO_2_ levels (380 ppm or 970 ppm). The selected salinity levels were based on the salinity that the barnacles were collected from and reared in (30-33 PSU), as well as the salinity of the central parts of the Baltic Sea (6 PSU), where the barnacle *B. improvisus* also is found. The selected pCO_2_ levels were based on ambient CO_2_ concentrations today (380 ppm) as well as the predictions by the Intergovernmental Panel on Climate Change (IPCC) climate models for year 2100 (970 ppm) [1]. The low salinity treatment was obtained by mixing 33 PSU of sterile filtered seawater with water filtered by reverse osmosis. The control CO_2_ treatment was determined by bubbling ambient air (with approx. 380ppm pCO_2_) in 5 L bottles of seawater of both 33 and 6 PSU until saturation was reached and pH was stabilized. The same procedure was performed for the high CO_2_ concentration using a gas cylinder with pre-mixed air with pCO_2_ levels of 970 ppm (AGA). The combination of the two different salinities and two saturating pCO_2_ levels resulted in four different pH levels. pH computers (Aqua Medic pH computer, Aqua Medic AB GmbH) were used to control the mixture of air and CO_2_ coming into the system to keep the pH stable at these four levels throughout the experiment. Approximately 300-800 larvae were placed in four 2L bottles containing each of the treatments. The experiment was run at a constant temperature of 21 +/- 0.5°C for 24 h. Visual inspection showed that the cyprids were healthy and swimming after the 24 hours in the different treatments and there was no difference in the amount of dead cyprids in any of the treatments. Even after longer incubations the mortality of cyprids in all treatments was low, reaching a maximum of 28% after 51 days in the treatment with low pCO_2_ and low salinity. There was no significant effect of salinity or pCO_2_ treatments on settlement. For expression studies, the larvae were filtered through a 10µm mesh, quickly transferred to cryotubes, and then immediately frozen in liquid nitrogen and placed in -80 °C. The experimental procedure above was repeated four times with different batches of larvae.

### Statistical analysis of NAK expression

Differences in NAK expression between soma and cirri were analyzed using paired t-tests, and between adult and cyprid using unpaired t-test with unequal variances. Differences in expression of the long and short NAK isoform within tissues/stages (i.e. within soma, cirri, adults or cyprids) were analyzed using paired t-tests. The effects of salinity and pCO_2_ on NAK expression in cyprids were analyzed using Analysis of Variance (ANOVA). Differences between treatment groups were tested *post-hoc* using Tukey’s test.

### Genome sequencing of B. *improvisus*

Genomic DNA was prepared from one individual of *B. improvisus* as previously described [73] and sent to SciLife Lab Stockholm for paired-end Illumina sequencing (100 bp read length) of both 150 bp and 300 bp fragment libraries. The reads were filtered and trimmed using the FASTX-Toolkit (http://hannonlab.cshl.edu/fastx_toolkit/). Assembly of the sequence reads into contigs was made using the CLC de novo assembler v4.06beta.67189 (www.clcbio.com). Contigs matching the experimentally derived NAK mRNA and DNA sequences were retrieved using the NCBI BLAST package. Exon-intron borders were further analyzed using GeneWise [78]. 

### Phylogenetic analysis

Phylogenetic trees were constructed from multiple alignments of N-terminally truncated NAK sequences from *B. improvisus* and from other organisms e.g. humans, zebrafish, insects, crustaceans and other invertebrates, as well as choanoflagellates. As outgroup, the *Dictyostelium* P-type ATPases most similar to metazoan Na^+^/K^+^ ATPases were used. In addition, human H^+^/K^+^ ATPases, which belong to the same group of P-type ATPases, were included in the analysis. The program Mr Bayes (v 3.2) was used with mixed amino-acid substitution matrices and run for 10,000,000 generations at which time the standard deviation was stable around 0.025. Analysis of the sample statistics showed that the mixing of the MCMC-chains was adequate.

For the *B. amphitrite* NAKs, NAK1 and NAK2 mRNA contigs were constructed with Cap3 by using all *B. amphitrite* EST libraries in both the Short Read Archive (SRA) and nucleotide databases at NCBI [32,33]. Resulting contigs were compared with cloned NAK sequences from *B. improvisus* and corrected if necessary.

### Correction of the N-terminus of the *Daphnia pulex* NAK (EFX88361) and *Monosiga brevicollis* NAK (XP_001742517)

For the *Daphnia* NAK protein EFX88361.1, we used an alternative Joint Genome Institute (JGI) gene prediction for locus JGI_V11_305634 (hxAUG26up1s4g197t1) that contained an exon encoding the 27 amino acids, which is presently annotated as a UTR. This new gene prediction is used in the 2010.4 draft of the *Daphnia* genome. Comparison of NAK sequences from the choanoflagellates *Monosiga brevicollis and Salpingoeca rosetta* indicated that the *M. brevicollis* (M.bre) XP_001742517 protein might lack the N-terminus, and therefore the upstream region of the gene was searched using a conserved part of the 27 amino acid stretch (RSESYRNAV) from one of the *S. rosetta* (S.ros) NAKs. The corresponding region found in *M. brevicollis* was used in the alignment of the 27 amino acid region with the conserved DSYR motif (Figure 9).

### Accession numbers for the NAK1-L, NAK1-S and NAK2 mRNA sequences

The *Balanus* NAK1-L, NAK1-S and NAK2 mRNA sequences have been deposited to GenBank and have the accessions number KC357568, KC357569 and KC357570 respectively.

## Supporting Information

Appendix S1 **Supplemental material and methods for cloning and PCR.** Description of cloning and PCR procedures.
(PDF)

Figure S1 **Expression of qPCR reference genes in tissues and life-stages.** **A**) Expression of the five qPCR reference genes actin, RPL8, NADHd1, EF1, 36B4 in two different life stages (cyprid and adult) and two different tissues (soma and cirri) is shown as
E^-Ct^, where E is primer efficiency and Ct is the qPCR cycle threshold value. In total 43 different samples were used in this analysis, consisting of 14 soma and 14 cirri from the same 14 individuals, 10 adults (soma plus cirri, excluding mantle or ovary tissue) and 5 cyprid batches (~1,000 individuals per batch). Error bars show the standard deviation. **B**) The coefficient of variation (CV: standard deviation/average) for the different references genes from the data in A is shown. **C**) To compare the expression pattern of the different reference genes, their expression was normalized to the expression of the soma in the adult.
(TIF)

Figure S2 **Pairwise correlation of the qPCR reference gene expression.** The pairwise correlation of the Ct values for the five reference genes is shown; in total there are 10 pair-wise comparisons. In each case the linear correlation coefficient (R^2^) is indicated and is in the range 0.33-0.80. The different sample types (soma, cirri, adults and cyprids) are color-coded for detection of any over- or under-expression of one gene compared to the other in any of the sample groups. No such differences in expression are obvious.
(TIF)

Figure S3 **Determination of the optimum number of reference genes.** The geNorm pairwise variation V (Vn/Vn+1) is calculated between the two sequential normalization factors NFn and NFn+1 for all the samples included in the analysis. It indicates whether the inclusion of an extra reference gene adds stability to the normalization factor. The geNorm pairwise variation V with successive inclusion of the less stable reference genes is shown. For geNorm stability values see Table S4. The pairwise variation V is lowest with inclusion of all five reference genes and is below the recommended value of 0.15.
(TIF)

Figure S4 **Expression of qPCR reference genes in cyprids exposed to different salinities and pCO_2_ levels.** Expression of the five qPCR reference genes RPL8, NADHd1, EF1, actin and 36B4 were analyzed in cyprids after exposure for 24 hours to pair-wise combinations of salinities of 33 PSU or 6 PSU and pCO_2_ levels of 970 or 380 ppm. **A**) Expression of the five reference genes is shown as E^-Ct^, where E is primer efficiency and Ct is the qPCR cycle threshold value. Error bars show the standard deviation. **B**) The coefficient of variation (CV: standard deviation/average) for the different treatments in A is shown. **C**) The expression of the reference genes was normalized to the expression in the treatment of 33 PSU salinity and 380 ppm pCO_2_. All reference genes had a tendency for lower expression in the low salinity treatments.
(TIF)

Figure S5 **Expression of *NAK1* and *NAK2* in response to various salinities and pCO_2_ levels.** QPCR was performed to measure the expression of the long and short NAK1 splice variants, as well as the total NAK1 and NAK2 expression, in a batch of cyprid larvae. Cyprids obtained from the Swedish west-coast were exposed for 24 hours to pair-wise comparisons of salinities of 33 PSU or 6 PSU and pCO_2_ levels of 970 or 380 ppm. The average expression in four independent cyprid batches is shown for each treatment. Expression is shown as E^-Ct^ where Ct is the cycle threshold *value and E* *is* *the* *primer* *efficiency*. No normalization to control genes was performed, since they exhibited a systematic decrease in the low salinity treatment. Error bars show the standard deviation. *A*) *No significant differences were found for the long (ANOVA, P=0.32) or the short (ANOVA, P=0.061) NAK1 splice variants*. Note that the relative expression of the long and short splice variants showed a significant change for the low salinity treatment (see Figure 8). *B*) *No* *significant* *differences* *in* *NAK1* *mRNA* *expression* *between* *treatments* *were* *found* (ANOVA, *P*=0.708). *C*) *No* *significant* *differences* *in* *NAK2* *mRNA* *expression* *between* *treatments* *were* *found* (ANOVA, *P*=0.072).
(TIF)

Table S1 **Primers for cloning and QPCR.** Name and sequence for the primers used in this study. All primers are written in the 5´ to 3´direction.
(PDF)

Table S2 **PCR programs for cloning of the *B. improvisus* Na+/K+ ATPases (NAKs).** The PCR programs used in the RACE reactions and during the cloning of the full-length NAKs are displayed. Programs P1 is a touchdown PCRs according to the GeneRacerTM kit manual (Invitrogen), where the annealing step of 65° C is excluded in the first ten 10 cycles. The first 5 cycles are run with a combined elongation/annealing step at 72° C, followed by 5 cycles of 70° C. The next 25 cycles are run with an annealing step at 65° C and an elongation temperature of 72° C.
(PDF)

Table S3 **Sequences used in Figure 5 and Figure 9.**
(XLSX)

Table S4 **Estimation of the stability of reference genes using geNorm, NormFinder and BestKeeper.** In geNorm and NormFinder a low stability value indicates a more stable gene, whereas in BestKeeper the coefficient of correlation should be as close to one as possible for a stable gene [1-3]. Recommended M values for geNorm are M<0.5 for homogeneous samples and M<1 for more heterogenous samples [4].
1. Vandesompele J, De Preter K, Pattyn F, Poppe B, Van Roy N, et al. (2002) Accurate normalization of real-time quantitative RT-PCR data by geometric averaging of multiple internal control genes. Genome Biol 3: RESEARCH0034.
2. Andersen CL, Jensen JL, Orntoft TF (2004) Normalization of real-time quantitative reverse transcription-PCR data: a model-based variance estimation approach to identify genes suited for normalization, applied to bladder and colon cancer data sets. Cancer Res 64: 5245-5250.
3. Pfaffl MW, Tichopad A, Prgomet C, Neuvians TP (2004) Determination of stable housekeeping genes, differentially regulated target genes and sample integrity: BestKeeper--Excel-based tool using pair-wise correlations. Biotechnol Lett 26: 509-515.
4. Hellemans J, Mortier G, De Paepe A, Speleman F, Vandesompele J (2007) qBase relative quantification framework and software for management and automated analysis of real-time quantitative PCR data. Genome Biol 8: R19.
(PDF)
