# Supplementary material for: Molecular Characterization of the α-Subunit of Na+/K+ ATPase from the Euryhaline Barnacle Balanus improvisus Reveals Multiple Genes and Differential Expression of Alternative Splice Variants
Source: PLoS One. 2013 Oct 9;8(10):e77069. doi: 10.1371/journal.pone.0077069 (PMC3793950; doi:10.1371/journal.pone.0077069)
Supplement: Appendix S1 — Supplemental material and methods for cloning and PCR. Description of cloning and PCR procedures. (PDF) [file pone.0077069.s001.pdf]

Appendix S1

Supplemental material and methods for cloning and PCR

Cloning of Na^+/K^+ atpase 1 from cDNA

To clone the *NAK1* cDNA from *B. improvisus*, 5´and 3´ RACE was performed with primers complementary to NAK EST sequences obtained from a *B. improvisus* cDNA Library (Alm Rosenblad et al., unpublished). The GeneRacer™ kit (Invitrogen) was used. For the 5´ RACE, primer NAK1_5´_3 was used together with GeneRacer 5´ primer (provided with the kit) and for 3´RACE, primer NaK1_3´_4 and NaK1_3´_5 was used together with the GeneRacer 3´ primer (provided with the kit). The touchdown PCR program P1 was applied. Table S1 contains all primer sequences used and Table S2 shows all PCR programs applied. The template for the PCR reaction was 75 ng RACE cDNA obtained from a pool of approximately 1000 cyprids. Nested PCR was then performed on 1 µl from the initial PCR reaction with the NaK1_5´_2 and NaK1_5´_1 primer together with the GeneRacer 5´ nested primer for the 5´RACE products and with the NaK1_3´_3 primer together with the GeneRacer 3´ nested primer for the 3´RACE products. The nested PCR was run according to program P2 and the polymerase PfuUltra High-Fidelity DNA polymerase (Stratagene) was used in the reactions. The PCR fragments were cloned into pCR®4Blunt-TOPO® using the Zero Blunt® TOPO® PCR Cloning Kit for Sequencing (Invitrogen). Six clones from the 5´RACE and three clones from the 3´RACE were sequenced. Interestingly, in the 5´RACE products, four different lengths of the extreme N-terminus of the predicted open reading frame were found. The variable N-terminal parts consisted of 31, 29, 4 and 2 amino acid (clones named accordingly N31, N29, N4 and N2), respectively. The difference between the four different N-termini is the inclusion or occlusion of a 27 amino acid that is preceded by either two or four amino acids at the predicted translation start.

In an attempt to obtain the full-length NAK, PCR was performed with primers complementary to sequences before the predicted start ATG in the 5´RACE products and after the predicted stop in the 3´RACE products. The polymerase PfuUltra was used in the reaction. However, no fragment was obtained. PCR primers were then designed to amplify parts of the NAK cDNA from just before the ATG (primer NaK1_fw) to 440 bases upstream of the stop (NaK1_rev_m) and from around 650 bp after the ATG (NaK1_fw_m) to just after the stop (primer NaK1_rev). PCR was run using Program P3. The fragments obtained were

cloned using Zero Blunt® TOPO® PCR Cloning Kit for Sequencing and three clones for each PCR fragment were sequenced. Out of the three clones spanning the start ATG, two had 5´ends similar to the N31 clone obtained in the 5´ RACE and one had a 5´end similar to the N2 clone. In order to amplify a fragment containing the whole open reading frame, spanning from before the predicted ATG to after the stop, the polymerase High Expand Fidelity^{plus} (Roche) was used with the primers NaK1_fw and NaK1_rev applying program P4. Fragments of around 3000 bp containing the full open reading frame were obtained. Two clones were sequenced and shown to encode for proteins of 1044 and 1017 amino acids. The clones were very similar except for a 27 amino acid difference in the N-terminus and their N-termini resembled the N4 and N31 clones in the 5´RACE. Thus, from the 5´RACE, the partial clones and the full-length clones, we conclude that Nak1 in *B.improvisus* is represented by a protein with four different lengths (1044, 1042, 1017 and 1015 amino acids).

Cloning of sequences corresponding to the N-terminus of Na^+/K^+ ATPase 1 from genomic DNA

In order to investigate the N-terminus of Nak1 at the level of genomic DNA, PCR was performed on 100 ng cyprid genomic DNA with primers based on cDNA sequences before the predicted start ATG and on sequences downstream of the 81 nucleotides long stretch specific for the long N-terminal variant. PCR with primers fw2_bfg, and rev_afg using program P5 and the polymerase PfuUltra yielded a fragment of around 5 kb. The 5 kb band was very weak, so additional PCR reactions applying program P6 or P7 were run, using the polymerase Expand High Fidelity^{plus} with the forward primers fw2_bfg and fw4_bfg in combination with reverse primers rev2_afg and rev3_afg. More PCR product was obtained and the 5 kb fragments were cloned into pCR®4Blunt-TOPO® or pCR®4-TOPO® and 13 clones were fully or partially sequenced.

To be able to detect PCR fragments larger than 5 kb, PCR was run on genomic DNA from cyprids with Expand Long (Roche) DNA polymerase that can amplify fragments up 20 kb. The forward primers fw2_bfg, fw3_bfg and fw4_bfg, which are complementary to sequences just up-stream of the start ATG, were used together with the reverse primer rev_afg, which is complementary to sequences just downstream of the 81-nucleotide stretch specific for the long variant. PCR program P8 was applied. When using fw2_bfg and fw3_bfg, only a 5 kb band was obtained. However, when using the primer fw4_bfg, an additional 7.1 kb band was

obtained. The 5 and 7.1 kb fragments obtained with the fw4_bfg primer were cloned into pCR®4-TOPO® using the TOPO® TA Cloning® Kit (Invitrogen). Two clones of each fragment (5 and 7.1 Kb) were fully or partially sequenced.

PCR was also run on 100 ng genomic DNA from one single adult using two sets of primers. The first set consists of forward primers fw2_bfg, fw3_bfg and fw4_bfg, complementary to sequences just upstream of the start ATG, and the reverse primer rev_ing that is complementary to sequences within the 81 nucleotide stretch, specific for the long variant of NAK1. The second set of primers consists of a forward primer within the 81 nucleotide long stretch (fw_ing) and a reverse primer (rev_afg) complementary to sequences just downstream of the 81-nucleotide stretch. The polymerase Expand High Fidelity^{plus} was used and PCR was run using program P9 for the first set and P10 for the second set. Fragments of around 5 kb were obtained with all primer pairs of set 1. For primer set 2, fragments of around 1.4 and 1.8 kb were obtained. The fragments were cloned into pCR®4-TOPO® using the TOPO® TA Cloning® Kit. Thirteen clones containing the 5 kb fragments were sequenced, revealing two different sequences. Six clones for each of the 1.4 and 1.8 kb fragments, respectively, were sequenced. Two different sequences were found among the 1.4 kb clones, whereas among the 1.8 kb clones, only one sequence was found.

Cloning of Na^+/K^+ atpase 2

PCR with degenerate primers complementary to a conserved part of arthropod NAKs (Towle *et al.*, 2001) were run on 100 ng genomic DNA from cyprids using program P11 and the polymerase Truestart Taq DNA Polymerase (Fermentas). Two bands of approximately 1000 bp and 720 bp in size were obtained. TOPO® TA Cloning® and subsequent sequencing of three clones from the 1000 bp band and five clones from the 720 bp band revealed that the 1000 bp band was part of the *NAK1* gene containing an intron and the other was a new sequence that was shown, using BLAST, to resemble another NAK alpha subunit than the previously cloned Nak1. This NAK we called Nak2.

To clone *NAK2* from cDNA, 5´and 3´RACE were performed on RACE cDNA (70 ng) prepared from two single adults using the GeneRacer kit. Primer NaK2_5´_10 was used together with GeneRacer 5´ primer for 5´ RACE and primers NaK2_3´_4 and NaK2_3´_2 were used together with GeneRacer 3´ primer for the 3´ RACE. A PCR touchdown program

(P1) was run with the polymerase PfuUltra. A nested PCR was then performed on the 5´ RACE products with primers NaK2_5´_9 and NaK2_5´_7 together with the GeneRacer 5´ nested primer, using program P12. Obtained 5´RACE fragments were cloned into the pCR®4Blunt-TOPO® vector and four clones for each individual was sequenced. For the first individual, all clones had similar sequences and for the second individual two different sequences were found probably representing two alleles. For the 3´RACE, a nested PCR was run using the program P13 with primer NaK2_3´-1 together with GeneRacer 3´ nested primer. Five clones for each adult were sequenced and each individual was represented by two different sequences, probably representing two alleles of *NAK2*.

To clone the whole open reading frame encoding Nak2 from the two adult individuals, PCR with fw primers NaK2_fw1 and NaK2_fw6, complementary to sequences before the predicted start ATG in the 5´ RACE products, were used together with the reverse primer NaK2_rev_2 complementary to sequences after the predicted stop codon in the 3´ RACE products. PCR program P14 was run with the polymerase Expand High Fidelity^{plus}. For adult 1, forward primers complementary to sequences of the 5´RACE products of either adult 1 and adult 2 resulted in PCR products, whereas for adult 2 only the primer based on its specific 5´ RACE product resulted in an amplified fragment. Out of eight partially sequenced clones for adult 1 that were obtained with primer NaK2_fw1 and 4 clones obtained with NaK2_fw6, two sequence variants were found. Clones representing these sequence variants were fully sequenced. For adult 2, four partially sequenced clones contained two sequence variants and two clones representing these sequence variants were fully sequenced.

PCR to detect alternative intron/exon structures of the Na^+/K^+ ATPase1 N-terminus

Comparison of the coding part of the 5' end of the cDNA clones to the corresponding region of the genomic clones of *NAK1a* and *NAK1b* identified in this paper, suggests that the long and short protein variants are the result of alternative splicing including or excluding exon 2 consisting of 81 nucleotides. However, to exclude the possibility that the short cDNA variant could origin from a gene lacking exon 2, PCR were performed with three different forward primers and two different reverse primers. In addition, different DNA polymerases were tested. A gene lacking the 81 bp exon would be easily detected in the PCR if the size of the fragment was smaller than the 7 or 4.7 kb that we obtained for *NAK1b* and *NaK1a*, respectively. If no intron exists between exon 1 and exon 3, a very short fragment of around

200 bp would have been produced with the primer pairs used. Performing PCR with primer pairs fw3_bfg and rev_afg, fw3_bfg and rev2_afg and fw4_bfg and rev2_afg using the polymerase PfuUltra gave bands of various size shorter than 5 kb, but larger than 200 bp. However, after cloning and sequencing of the amplified fragments all were shown to be non-specific products not related to NAK. PCR was also performed using the polymerase Expand Long, which is used to obtain large fragments. Conditions favoring amplifying fragments of 20 kb size were used according to the manufacturer's protocol. Performing PCR on 100 ng genomic DNA from a batch of cyprids with the forward primers fw2_bfg, fw3_bfg and fw4_bfg in combination with the reverse primers rev_ afg and rev2_ afg in 6 separate reactions, yielded one 4.7 kb band for the primers fw2_bfg and fw3_bfg in combination with both reverse primers, whereas fw4_bfg with both reverse primers yielded the same 4.7 kb fragment and an additional 7 kb fragment. All these fragments were after cloning and sequencing shown to be specific NAK bands. No other bands were present in the gel. It was therefore concluded that unless there is a really big intron (resulting in a PCR fragment larger than 7.1 kb) connecting exon 1 and exon 2, the short and long variants of the cDNA we cloned are the result of alternative splicing of one gene.

PCR on the genomic DNA encoding the Nak2 N-terminus

In order to investigate the Nak2 N-terminus at the level of genomic DNA, PCR was performed on two adults with the forward primers NaK2_fw1 and NaK2_fw6 and the reverse primers NaK2_5´_9 and NaK2_5´_6. PCR program P3 was used. Sequencing showed that several clones contained sequences from unspecific PCR products. However, three clones from adult 1 and two clones from adult 2 contained *NAK2* sequences and revealed an intron just after the start methionine, with an average size of 1 kb.

Quantitative real-time PCR analysis of differential gene expression

All qPCR reactions were performed using SYBR Green supermix (Bio-Rad). The PCR was performed with an initial denaturation temperature of 95°C for 3 min, a denaturation step at 95°C for 20 s, an annealing temperature of 58-60°C for 20 seconds and elongation at 72°C for 30 seconds. In total 40 PCR cycles were run.

The forward primers fw_IsoS and fw_IsoL were designed to specifically pick up the short and long cDNA variants of *NAK1* respectively (containing or not containing an 81 nucleotide insertion in the 5´end). Two long variants exist, where the 81 nucleotide insertion is preceded by either 12 or 6 nucleotides, which codes for the first four or two amino acids of the protein. There are also two short variants lacking the 81 nucleotide insertion, which contain the same 12 or 6 nucleotides as the long variant at the translation start site. It was not possible to make primers selecting between the two shorter variants, differing only by six nucleotides. The primer specific for the short variant (fw_IsoS) has its 5´end before the 81 nucleotides insertion (starting at ATG) and its 3´end just after the insertion in the part common for the long and short variants. The forward primer for the long variant (fw_IsoL) is located within the 81-nucleotide sequence specific for the long variants and thus does not distinguish between the two 5´end forms. The specificity of the primers was checked by running qPCR at an annealing temp of 56°C, using plasmids containing the different constructs (in a range of concentrations from 0.0015 to 12.5 ng). The primer specific for the short variants was shown to amplify the short variants at Ct values 12-14 lower than the longer variants and to amplify the two short variants (differing by six nucleotides) equally well.

To compare expression differences of *NAK1*, *NAK2* and the long and short variants of *NAK1* in different life stages and tissues, qPCR was performed on five batches of cyprids, 10 adults (soma and cirri, mantle and ovary tissue excluded) and in the soma and cirri from 14 adults. For *NAK1*, primers NaK1_fw3_Q and NaK1_rev1_Q was used at an annealing temperature of 58°C. For *NAK2*, NaK2_fw2_Q and NaK2_rev2_Q was used at an annealing temperature of 59°C. For the short and long variants of *NAK1*, the forward primers fw_IsoS and fw_IsoL together with the reverse primer rev_Iso, at an annealing temperature of 58°C. 1 ng cDNA was used in the reactions.

qPCR was performed on four batches of cyprids exposed to combinations of two different pCO_2 pressures and two different salinities. We first used 1.4 ng cDNA with forward primers fw_IsoS and fw_IsoL together with the reverse primer Iso_rev, at an annealing temperature of 58°C. qPCR on the same batches of cyprids was then performed on 4 ng cDNA with forward and reverse primers complementary to sequences in the 3´end of *NAK1* (NaK1_fw3_Q and NaK1_rev1_Q), thus picking up both the long and short 5´end variants, as well as with primers that were specific for NAK2 (NaK2_fw2_Q and NaK2_rev2_Q). Annealing temperatures of 58°C and 60°C were used in the first and second qPCR, respectively.

Primers for the five reference genes actin, RPL8 (ribosomal protein L8), EF1 (Elongation factor alpha 1) and NADHd1 (NADH dehydrogenase subunit 1) and 36B4 (RPLP0, Ribosomal protein P0) were designed using EST sequences obtained from a *B. improvisus* cDNA Library (Alm Rosenblad et al., unpublished). qPCR was run on the life-stage and tissue samples as well as on the salinity and pCO_2 treated samples described above using primers Actin_fw with Actin_rev; RPL8_fw1 with RPL8_rev1; EF1_fw1 with EF1_rev1; NADHd1_fw2 with NADHd1_rev2 and 36B4_fw2 with 36B4_rev2. An annealing temperature of 58°C was applied to all primers, except for EF1, which was run at 60°C. 1 and 1.4 ng cDNA were used for the life stage/tissue samples and salinity/pCO_2 treated cyprids, respectively.

References

Lind U, Alm Rosenblad M, Hasselberg Frank L, Falkbring S, Brive L, Laurila JM*, et al.* Octopamine receptors from the barnacle balanus improvisus are activated by the alpha2-adrenoceptor agonist medetomidine. *Mol Pharmacol* **78**(2)**:** 237-248.

Towle DW, Paulsen RS, Weihrauch D, Kordylewski M, Salvador C, Lignot JH*, et al.* (2001). Na++K+-ATPase in gills of the blue crab Callinectes sapidus: cDNA sequencing and salinity-related expression of alpha-subunit mRNA and protein. *Journal of Experimental Biology* **204**(22)**:** 4005-4012.
