# Supplementary figures and images for: Molecular Characterization of the α-Subunit of Na+/K+ ATPase from the Euryhaline Barnacle Balanus improvisus Reveals Multiple Genes and Differential Expression of Alternative Splice Variants
Source: PLoS One. 2013 Oct 9;8(10):e77069. doi: 10.1371/journal.pone.0077069 (PMC3793950; doi:10.1371/journal.pone.0077069)

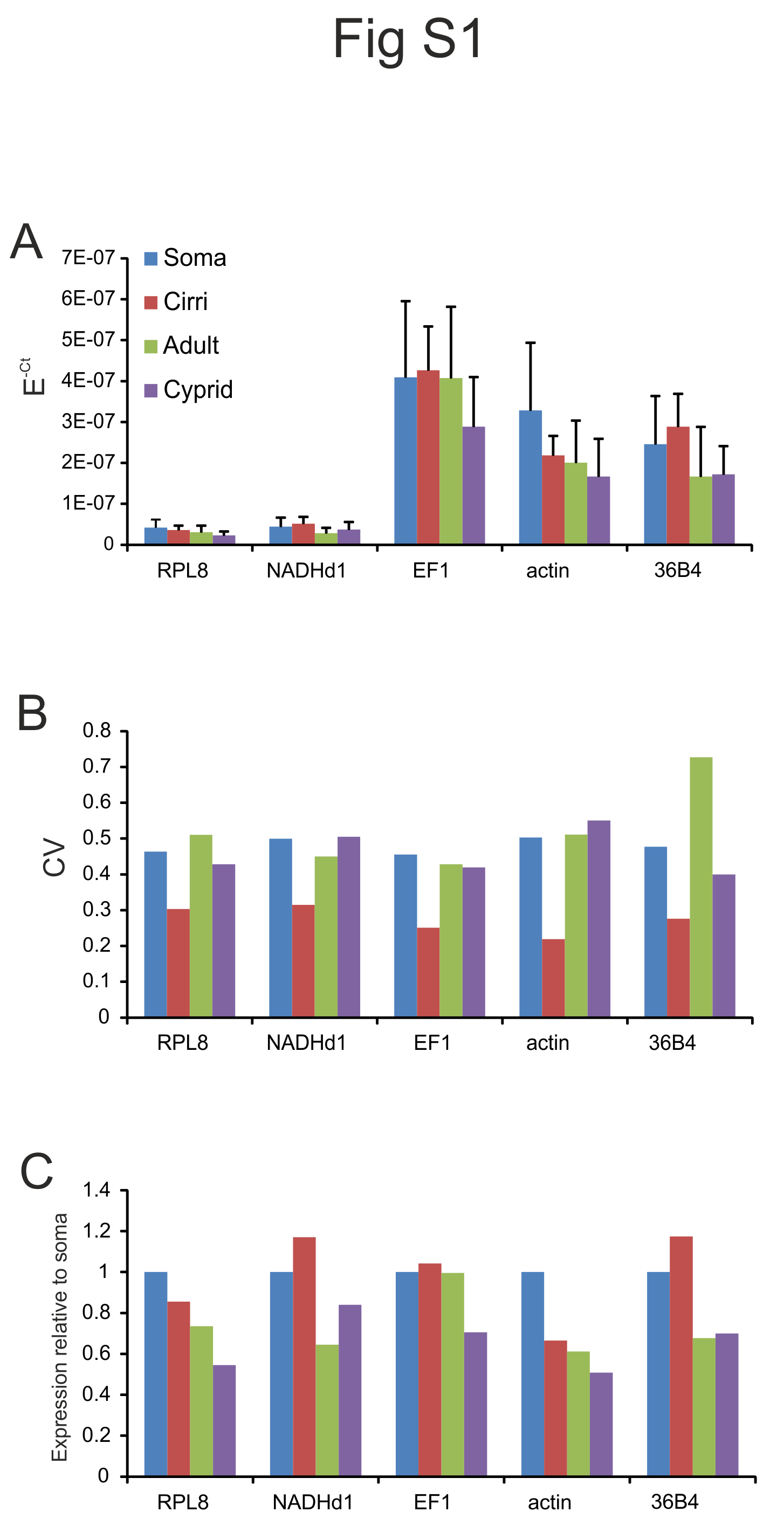

Supplement: Figure S1 — Expression of qPCR reference genes in tissues and life-stages. A) Expression of the five qPCR reference genes actin, RPL8, NADHd1, EF1, 36B4 in two different life stages (cyprid and adult) and two different tissues (soma and cirri) is shown as E-Ct, where E is primer efficiency and Ct is the qPCR cycle threshold value. In total 43 different samples were used in this analysis, consisting of 14 soma and 14 cirri from the same 14 individuals, 10 adults (soma plus cirri, excluding mantle or ovary tissue) and 5 cyprid batches (~1,000 individuals per batch). Error bars show the standard deviation. B) The coefficient of variation (CV: standard deviation/average) for the different references genes from the data in A is shown. C) To compare the expression pattern of the different reference genes, their expression was normalized to the expression of the soma in the adult. (TIF) [file pone.0077069.s002.tif]

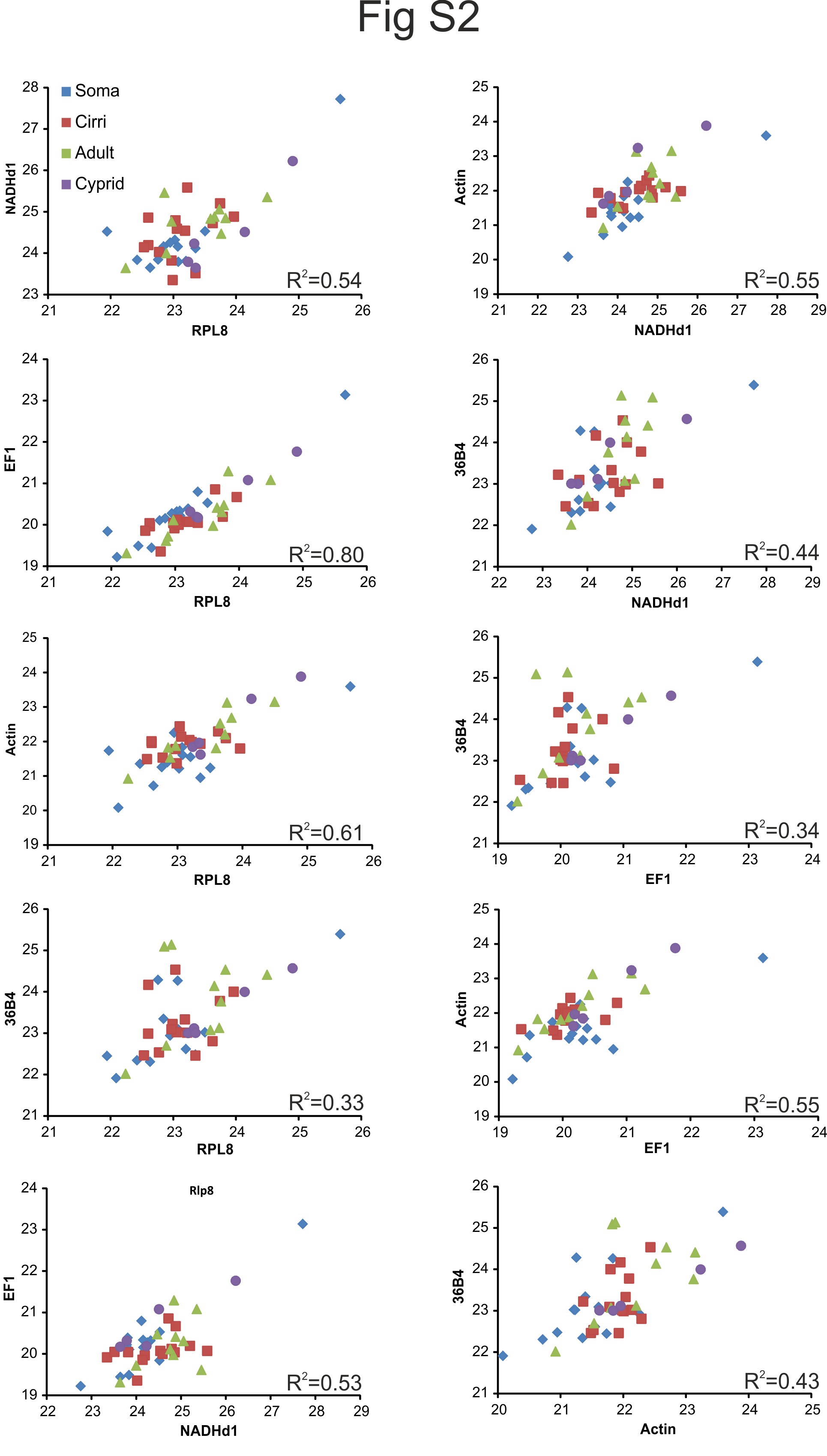

Supplement: Figure S2 — Pairwise correlation of the qPCR reference gene expression. The pairwise correlation of the Ct values for the five reference genes is shown; in total there are 10 pair-wise comparisons. In each case the linear correlation coefficient (R2) is indicated and is in the range 0.33-0.80. The different sample types (soma, cirri, adults and cyprids) are color-coded for detection of any over- or under-expression of one gene compared to the other in any of the sample groups. No such differences in expression are obvious. (TIF) [file pone.0077069.s003.tif]

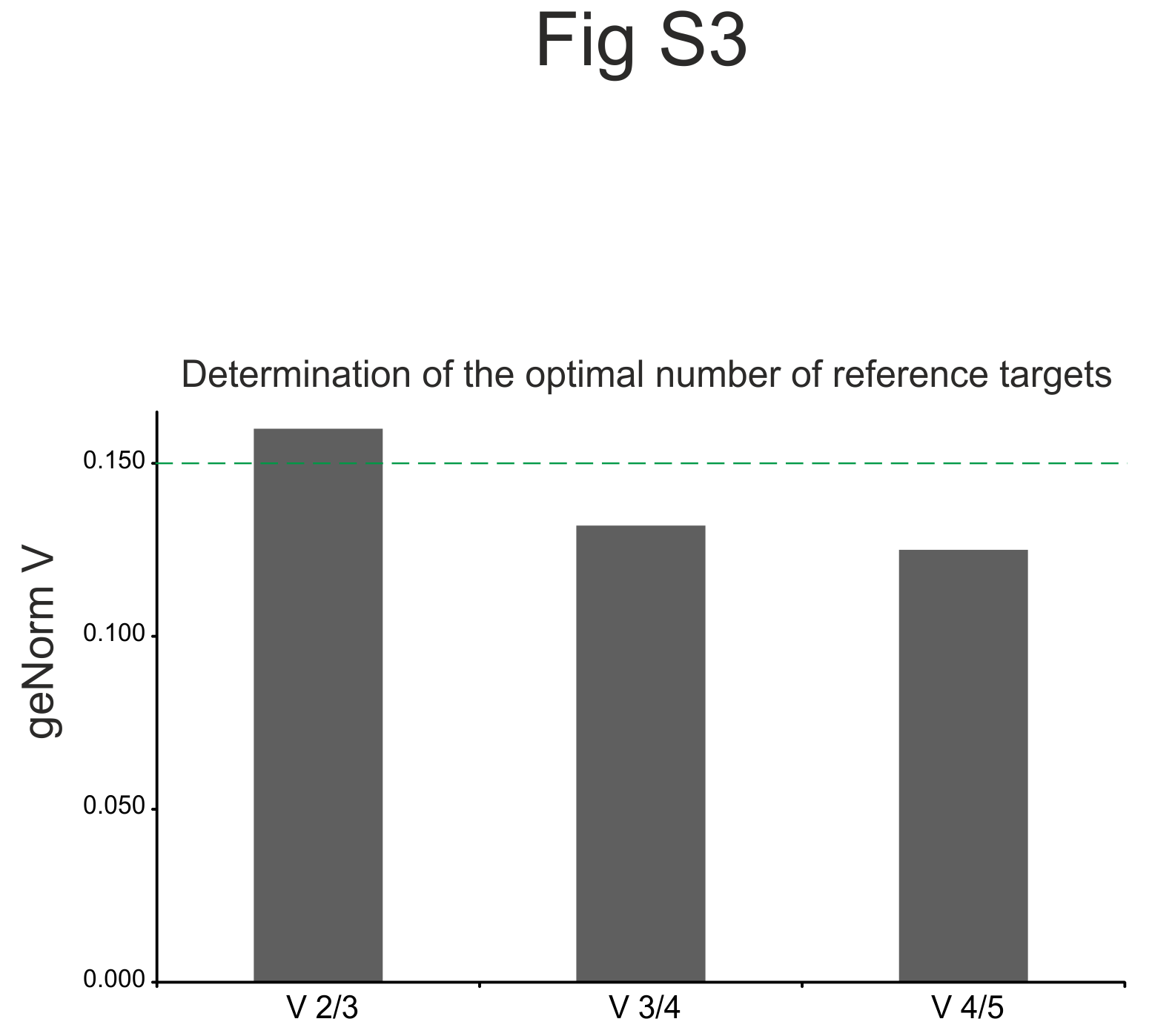

Supplement: Figure S3 — Determination of the optimum number of reference genes. The geNorm pairwise variation V (Vn/Vn+1) is calculated between the two sequential normalization factors NFn and NFn+1 for all the samples included in the analysis. It indicates whether the inclusion of an extra reference gene adds stability to the normalization factor. The geNorm pairwise variation V with successive inclusion of the less stable reference genes is shown. For geNorm stability values see Table S4. The pairwise variation V is lowest with inclusion of all five reference genes and is below the recommended value of 0.15. (TIF) [file pone.0077069.s004.tif]

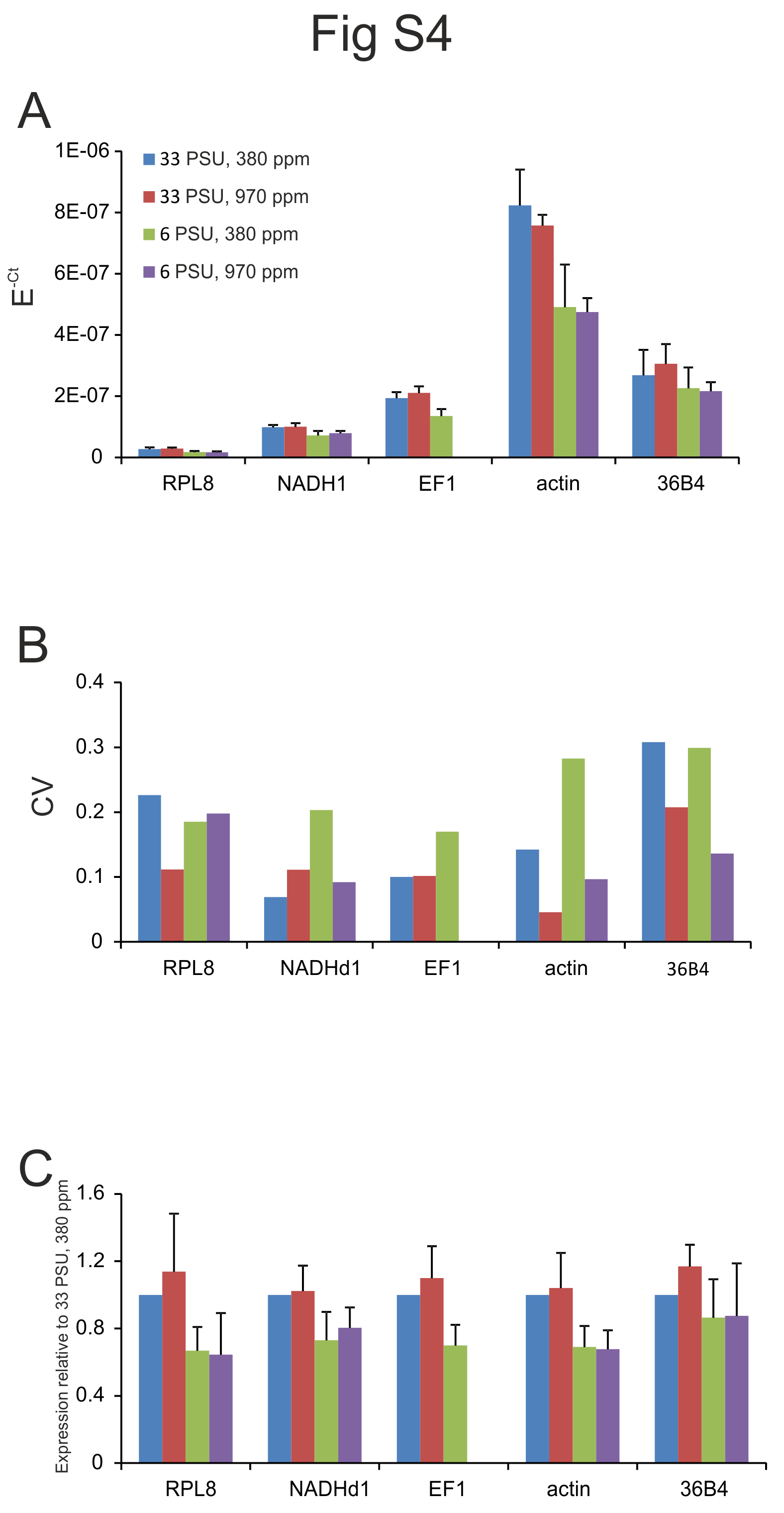

Supplement: Figure S4 — Expression of qPCR reference genes in cyprids exposed to different salinities and pCO2 levels. Expression of the five qPCR reference genes RPL8, NADHd1, EF1, actin and 36B4 were analyzed in cyprids after exposure for 24 hours to pair-wise combinations of salinities of 33 PSU or 6 PSU and pCO2 levels of 970 or 380 ppm. A) Expression of the five reference genes is shown as E-Ct, where E is primer efficiency and Ct is the qPCR cycle threshold value. Error bars show the standard deviation. B) The coefficient of variation (CV: standard deviation/average) for the different treatments in A is shown. C) The expression of the reference genes was normalized to the expression in the treatment of 33 PSU salinity and 380 ppm pCO2. All reference genes had a tendency for lower expression in the low salinity treatments. (TIF) [file pone.0077069.s005.tif]

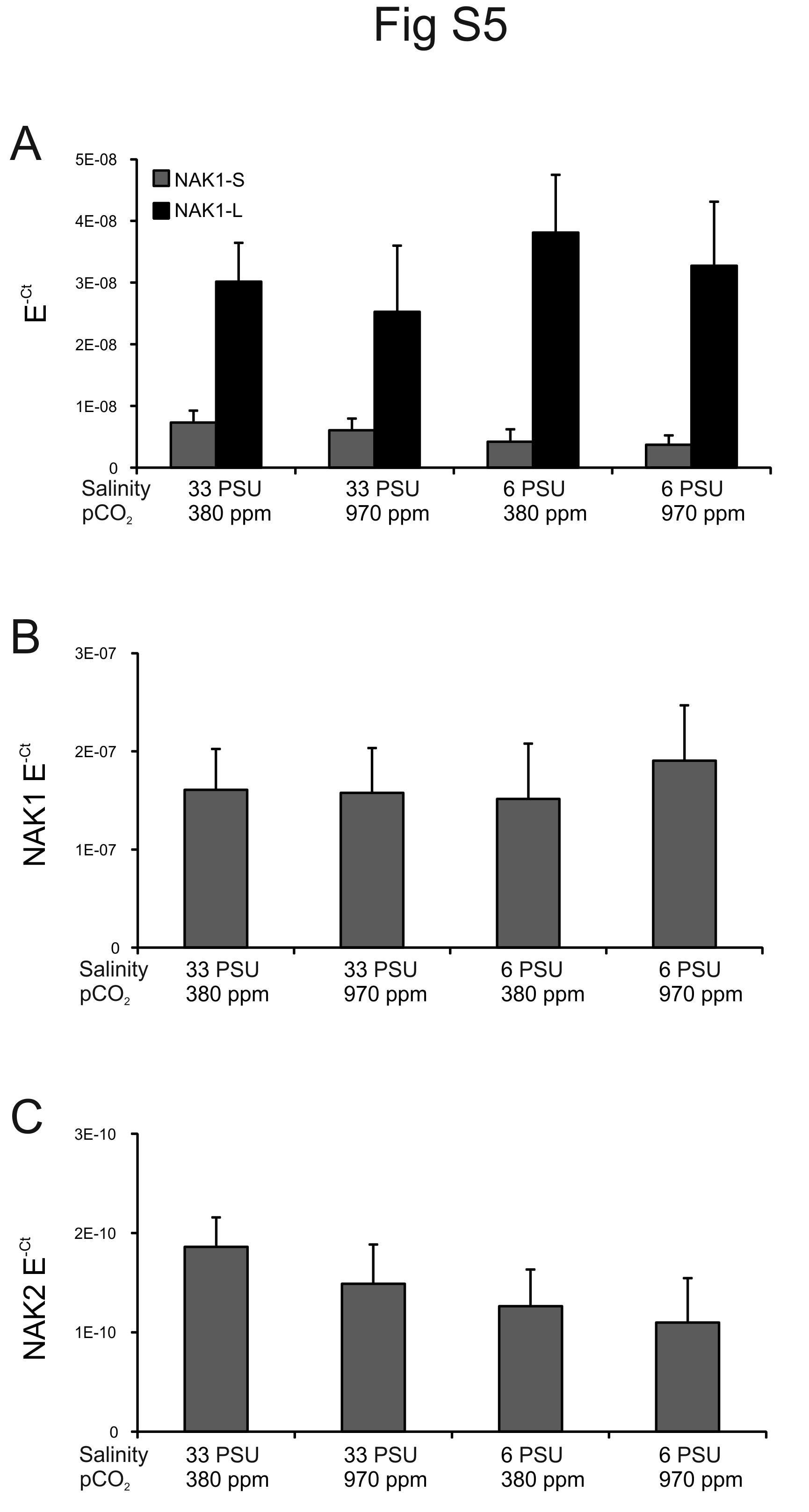

Supplement: Figure S5 — Expression of NAK1 and NAK2 in response to various salinities and pCO2 levels. QPCR was performed to measure the expression of the long and short NAK1 splice variants, as well as the total NAK1 and NAK2 expression, in a batch of cyprid larvae. Cyprids obtained from the Swedish west-coast were exposed for 24 hours to pair-wise comparisons of salinities of 33 PSU or 6 PSU and pCO2 levels of 970 or 380 ppm. The average expression in four independent cyprid batches is shown for each treatment. Expression is shown as E-Ct where Ct is the cycle threshold value and E is the primer efficiency. No normalization to control genes was performed, since they exhibited a systematic decrease in the low salinity treatment. Error bars show the standard deviation. A) No significant differences were found for the long (ANOVA, P=0.32) or the short (ANOVA, P=0.061) NAK1 splice variants. Note that the relative expression of the long and short splice variants showed a significant change for the low salinity treatment (see Figure 8). B) No significant differences in NAK1 mRNA expression between treatments were found (ANOVA, P=0.708). C) No significant differences in NAK2 mRNA expression between treatments were found (ANOVA, P=0.072). (TIF) [file pone.0077069.s006.tif]
