# Supplementary material for: Molecular Characterization of the α-Subunit of Na+/K+ ATPase from the Euryhaline Barnacle Balanus improvisus Reveals Multiple Genes and Differential Expression of Alternative Splice Variants
Source: PLoS One. 2013 Oct 9;8(10):e77069. doi: 10.1371/journal.pone.0077069 (PMC3793950; doi:10.1371/journal.pone.0077069)
Supplement: Table S1 — Primers for cloning and QPCR. Name and sequence for the primers used in this study. All primers are written in the 5´ to 3´direction. (PDF) [file pone.0077069.s007.pdf]

Table S1. Primers for cloning and QPCR. Name and sequence for the primers used in this study. All primers are written in the 5´ to 3´direction.

**Primer name**	**Primer sequence**
NaK1 _5´_1	GCGACGAGTTGTCCACCTTCATGTT
NaK1 _5´_2	GAGCGGGTTCTCGGCGGTGAACT
NaK1 _5´_3	CATGTTCCCTCGACGGCGTTGGT
NaK1 _3´_3	CGTACGGCCAGATCGGTATGATC
NaK1 _3´_4	GGCACCGTCACCATCCTCTGCAT
NaK1 _3´_5	CAAACTCGTCAACGAGAGGCTCATC
NaK1_fw	GCCGTTGCGACACCGAGTAAC
NaK1_rev	TGTGATGGCCGCGTGTCGTC
NaK1_fw_m	TGGTCGAGATCAAGTTCGGAGA
NaK1_rev_m	TTCCTGCCCTGGAACCTCGC
fw2_bfg	GGAGTGAAACCACCGACCATCAGA
fw3_bfg	CACAGCGCCACACCACCGGAGT
fw4_bfg	CTTCCGGCGGGAACCAACTGCAA
rev_afg	GCTCCTCGATGGTGATCATGTGCT
rev2_afg	GGGCATCATGCTTCTGACGCTTG
rev3_afg	TCCTGCTTGAGGTCGTCCAGGTT
fw_ing	ACGGTCGACCGGACTCGTACCG
rev_ing	CTTGAGCTGTCCGTGGGCTGTGA
NaK2_5´_7	CTGCGATGGGTATAAGATGCTCATC
NaK2_5´_9	CGGCATAGACACTGAAGCACAGAAT
NaK2_5´_10	GACATAGAAGTTGTCCGGCTCTACA
NaK2_3´_1	GACGCCTTTCCGGAGGGCTACA
NaK2-3´_2	TCTGCGACCTGGGACTGCCAGTG
NaK2-3´_4	CTACCACGGCGGCGACGTCAGT
NaK2_fw_1	CGCGTGGAAATCGACACACTGAC
NaK2_fw_6	GAGTTACAACAGGCACCCTTCGAC
NaK2_rev_2	ATCCAACCTTGAAAAGCAGCTCACA
fw_IsoS	ATGTCCATGGACTCCAAGC
fw_IsoL	CCCACGGACAGCTCAAGT
rev_Iso	CCTCGATGGTGATCATGTG
NaK1_fw3_Q	GTCCAGCAGGGCATGAACAAC
NaK1_rev1_Q	CGGCACTCGTCGTAGATGAAG
NaK2_fw2_Q	CTGAAGGACATGACGGAG
NaK2_rev2_Q	CAGCCTTCGACGATGATC
Actin_fw	CATCAAGATCAAGATCATCGC
Actin_rev	ATCTGCTGGAAGGTGGAC

36B4_fw2	TTGGACCCGAGAAGACTT
36B4_rev2	TACGAGAACGGAGAGATACC
NADHd1_fw2	AGCAGAAATGAATCGTACAC
NADHd1_rev2	AACCTCCAGCTCTATACTCA
EF1_fw1	GATGCACCACGAATCTCT
EF1_rev1	TTGACGGAGACGTTCTTCA
RPL8_fw1	ATACCAAGCACAGGAAAGG
RPL8_rev1	GTGGATCAGCTCCTTGAC
