# Supplementary material for: Molecular Characterization of the α-Subunit of Na+/K+ ATPase from the Euryhaline Barnacle Balanus improvisus Reveals Multiple Genes and Differential Expression of Alternative Splice Variants
Source: PLoS One. 2013 Oct 9;8(10):e77069. doi: 10.1371/journal.pone.0077069 (PMC3793950; doi:10.1371/journal.pone.0077069)
Supplement: Table S2 — PCR programs for cloning of the B. improvisus Na+/K+ ATPases (NAKs). The PCR programs used in the RACE reactions and during the cloning of the full-length NAKs are displayed. Programs P1 is a touchdown PCRs according to the GeneRacerTM kit manual (Invitrogen), where the annealing step of 65° C is excluded in the first ten 10 cycles. The first 5 cycles are run with a combined elongation/annealing step at 72° C, followed by 5 cycles of 70° C. The next 25 cycles are run with an annealing step at 65° C and an elongation temperature of 72° C. (PDF) [file pone.0077069.s008.pdf]

Table S2. PCR programs for cloning of the *B. improvisus* Na+/K+ ATPases (NAKs). The PCR programs used in the RACE reactions and during the cloning of the full-length NAKs are displayed. Programs P1 is a touchdown PCRs according to the GeneRacerTM kit manual (Invitrogen), where the annealing step of 65° C is excluded in the first ten 10 cycles. The first 5 cycles are run with a combined elongation/annealing step at 72° C, followed by 5 cycles of 70° C. The next 25 cycles are run with an annealing step at 65° C and an elongation temperature of 72° C.

**Program name**	**Initial denat.**	**Denat**	**Annealing**	**Elong**	**cycles**	**Final elong**
P1 (touchdown)	98°C, 2 min	98° C, 30 s	65° C, 30 s	70° C, 72° C	35 (5+5+25)	72° C, 10 min
			Excluded in the 10 first cycles	72° C, 2 min		
P2	98°C, 2 min	98°C, 30 s	66°C, 30 s	72°C, 2 min	35	72°C, 10 min
P3	98°C, 2 min	98°C, 30 s	59°C, 30 s	72°C, 2.5 min	35	72°C, 10 min
P4	94°C, 2 min	94°C, 30 s	61°C, 30 s	68°C, 3.5 min	35	68°C, 10 min
P5	98°C, 2 min	98°C, 30 s	63°C, 30 s	72°C, 2 min	35	72°C, 10 min
P6	94°C, 2 min	94°C, 30 s	58°C, 30 s	68°C, 4 min	35	68°C, 10 min
P7	94°C, 2 min	94°C, 30 s	59°C, 30 s	68°C, 4 min	35	68°C, 10 min
P8	94°C, 2 min	94°C, 30 s	58°C, 30 s	68°C, 8 min	35	68°C, 7 min
P9	94°C, 2 min	94°C, 30 s	58°C, 30 s	68°C, 4 min	35	68°C, 10 min
P10	94°C, 2 min	94°C, 30 s	58°C, 30 s	72°C, 4 min	35	72°C, 10 min
P11	95°C, 2 min	95°C, 60 s	45°C, 60 s	72°C, 2 min	33	72°C, 10 min
P12	98°C, 2 min	98°C, 30 s	61°C, 30 s	72°C, 2 min	35	72°C, 2 min
P13	98°C, 2 min	98°C, 30 s	60°C, 30 s	72°C, 2.5 min	35	72°C, 2 min
P14	94°C, 2 min	94°C, 30 s	58°C, 30 s	68°C, 3.5 min	35	68°C, 10 min
