# Supplementary material for: Molecular Characterization of the α-Subunit of Na+/K+ ATPase from the Euryhaline Barnacle Balanus improvisus Reveals Multiple Genes and Differential Expression of Alternative Splice Variants
Source: PLoS One. 2013 Oct 9;8(10):e77069. doi: 10.1371/journal.pone.0077069 (PMC3793950; doi:10.1371/journal.pone.0077069)
Supplement: Table S4 — Estimation of the stability of reference genes using geNorm, NormFinder and BestKeeper. In geNorm and NormFinder a low stability value indicates a more stable gene, whereas in BestKeeper the coefficient of correlation should be as close to one as possible for a stable gene [1-3]. Recommended M values for geNorm are M<0.5 for homogeneous samples and M<1 for more heterogenous samples [4]. 1. Vandesompele J, De Preter K, Pattyn F, Poppe B, Van Roy N, et al. (2002) Accurate normalization of real-time quantitative RT-PCR data by geometric averaging of multiple internal control genes. Genome Biol 3: RESEARCH0034. 2. Andersen CL, Jensen JL, Orntoft TF (2004) Normalization of real-time quantitative reverse transcription-PCR data: a model-based variance estimation approach to identify genes suited for normalization, applied to bladder and colon cancer data sets. Cancer Res 64: 5245-5250. 3. Pfaffl MW, Tichopad A, Prgomet C, Neuvians TP (2004) Determination of stable housekeeping genes, differentially regulated target genes and sample integrity: BestKeeper--Excel-based tool using pair-wise correlations. Biotechnol Lett 26: 509-515. 4. Hellemans J, Mortier G, De Paepe A, Speleman F, Vandesompele J (2007) qBase relative quantification framework and software for management and automated analysis of real-time quantitative PCR data. Genome Biol 8: R19. (PDF) [file pone.0077069.s010.pdf]

Table S4. **Estimation of the stability of reference genes using geNorm, NormFinder and BestKeeper.** In geNorm and NormFinder a low stability value indicates a more stable gene, whereas in BestKeeper the coefficient of correlation should be as close to one as possible for a stable gene [1-3]. Recommended M values for geNorm are M<0.5 for homogeneous samples and M<1 for more heterogenous samples [4].

**Reference Gene**	**geNorm stability (M)**	**NormFinder stability***	**BestKeeper coefficient of correlation(r)**
RLP8	0.545	0.237	0.908
EF1	0.546	0.236	0.900
Actin	0.567	0.249	0.902
NADH1d	0.614	0.310	0.886
36b4	0.703	0.413	0.813

*Stability calculated without taking sample subgroups into consideration.

1. Vandesompele J, De Preter K, Pattyn F, Poppe B, Van Roy N, et al. (2002) Accurate normalization of real-time quantitative RT-PCR data by geometric averaging of multiple internal control genes. Genome Biol 3: RESEARCH0034.
2. Andersen CL, Jensen JL, Orntoft TF (2004) Normalization of real-time quantitative reverse transcription-PCR data: a model-based variance estimation approach to identify genes suited for normalization, applied to bladder and colon cancer data sets. Cancer Res 64: 5245-5250.
3. Pfaffl MW, Tichopad A, Prgomet C, Neuvians TP (2004) Determination of stable housekeeping genes, differentially regulated target genes and sample integrity: BestKeeper--Excel-based tool using pair-wise correlations. Biotechnol Lett 26: 509-515.
4. Hellemans J, Mortier G, De Paepe A, Speleman F, Vandesompele J (2007) qBase relative quantification framework and software for management and automated analysis of real-time quantitative PCR data. Genome Biol 8: R19.
